# Volatile Organic Compound Emissions From *Solidago altissima* Under Experimental Warming and Drought

**DOI:** 10.1002/ece3.72422

**Published:** 2025-11-11

**Authors:** Kara C. Dobson, Phoebe L. Zarnetske

**Affiliations:** ^1^ Department of Integrative Biology Michigan State University East Lansing Michigan USA; ^2^ Ecology, Evolution, and Behavior Program Michigan State University East Lansing Michigan USA

## Abstract

Plant volatile organic compound (VOC) emissions are important mediators for plant interactions with biotic and abiotic factors in the environment. Changes in VOC emissions can be caused by factors associated with climate change, such as warming and drought. However, we currently lack an understanding of how warming and drought affect plants' emissions in their natural environment, let alone how these climate factors may interact to synergistically affect emissions. To fill these knowledge gaps, we measured VOC emissions from tall goldenrod ( *Solidago altissima* ) in an early successional plant community under four climate treatments: ambient control, warmed, drought, and warmed + drought. Treatments were applied in situ using open‐top chambers for warming and rainout shelters for drought. Drought treatments (drought and warmed + drought) have a stronger effect on VOC emissions compared to nondrought treatments (ambient and warmed). Furthermore, while the overall abundance of VOCs did not differ between treatments, there were specific compounds associated with one or more climate treatments. For example, diisopropyl adipate was more abundant in the drought and warmed + drought treatments. Our study shows that in goldenrod, drought may have a stronger effect than warming on VOC emissions, but moreover, that specific compounds are especially sensitive to certain climate treatments. However, additional experimentation is necessary to validate the functions associated with the affected compounds. These findings demonstrate that climate change alters chemical emissions, which in turn could have implications for ecosystem functioning via changes in plant–plant communication, plant–insect interactions, and overall plant fitness.

## Graphical abstract

Drought has a greater impact than warming on the VOC emissions of *Solidago altissima* . Additionally, a few specific compounds are particularly affected by these climate treatments, which may influence plant functioning through changes in stress protection mechanisms.

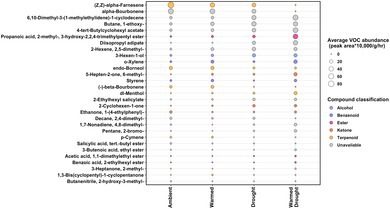

## 1 Introduction

Within an environment, plants face numerous multifaceted interactions with biotic and abiotic factors. Plant emissions of volatile organic compounds (VOCs) are important mediators in these interactions. For example, VOCs can serve as plant growth regulators, pathogen growth inhibitors, defense priming signals, and more (Brilli et al. 2019). However, it is currently unclear how climate change factors, such as warming and drought, may impact the emission of VOCs for plants. While previous studies have shown that warming and drought can change both the abundance and composition of emitted VOCs (Reinecke et al. 2024; Sharkey and Singsaas 1995; Peñuelas and Llusià 2003; Sharkey et al. 2007; Kreuzwieser et al. 2021), these changes can be dependent upon the severity of the stress experienced by the plant (Niinemets 2010; Rissanen et al. 2022) and the species that were sampled (Müller and Junker 2022), therefore, we lack an understanding of how these climate factors may impact VOC emissions for many plant species. Here, we seek to understand the effects of experimental warming and drought treatments on the foliar emissions of VOCs from a common herbaceous species in the midwestern United States, *Solidago altissima* .

Changes in VOC production can occur as a result of altered abiotic conditions, such as warming or drought. Typically, warming leads to an overall increase in foliar VOC production due to increased plant enzymatic activity, increased vapor pressure, and decreased resistance in the diffusion pathway (Hartikainen et al. 2009; Peñuelas and Llusià 2003; Yuan et al. 2009). Warming may also indirectly increase VOC emissions through increased plant biomass (Kramshøj et al. 2016). Conversely, drought can have varying effects on VOC emissions, and these differences may be due in part to the severity of the drought experienced by the plant. For example, moderate drought can lead to increased VOC production if limitation in growth leads to increased carbon availability, whereas a more severe drought can reduce photosynthesis and therefore reduce carbon allocation to VOC production (Reinecke et al. 2024; Rissanen et al. 2022; Malone et al. 2023; Tiiva et al. 2017; Niinemets 2010). Other climate‐induced limitations to the 2‐C‐methyl‐D‐erythritol 4‐phosphate (MEP) production pathway can reduce VOC production, such as changes in gene regulation of compound synthases (Perreca et al. 2022). However, there are currently still gaps in our understanding of VOC production under current climate regimes (Yuan et al. 2009), let alone under warmer and drier conditions; therefore, we lack complete understanding of these climate effects on VOC production. Furthermore, the majority of studies we identified in our literature search on the effects of warming and/or drought on VOC emissions focus on woody species (e.g., Rissanen et al. 2022; Kreuzwieser et al. 2021; Trowbridge et al. 2019; Staudt et al. 2016; Llusià et al. 2006), and to our knowledge, no study has used *Solidago* spp. as a focal species in climate‐related VOC experiments.

In addition to changes in overall VOC abundance, the composition of VOCs can also change in response to changes in abiotic conditions. For example, warming can increase the production of isoprene and monoterpenes, which may protect plants from heat stress (Ibrahim et al. 2006; Sharkey and Singsaas 1995; Sharkey et al. 2007). Similarly, Kreuzwieser et al. (2021) found that isoprene and monoterpene biosynthesis was maintained despite reduced photosynthesis due to drought, indicating that these compounds may have important functions for stress tolerance, such as quenching reactive oxygen species or stabilizing membranes (Loreto and Velikova 2001; Peñuelas and Llusià 2003; Velikova et al. 2011). Conversely, certain compounds or groups of VOCs could experience a reduction in emissions under stress if carbon is partitioned away from the production of those VOCs (Kreuzwieser et al. 2021). In sum, climate conditions can induce the production of novel compounds that aid in protection or prevent specific compounds from being produced. By identifying the compounds that are sensitive to climate conditions, we will be able to gain a better understanding of how plant emissions may be affected by climate change.

Climate warming and drought are projected to co‐occur more frequently in the future (IPCC 2021), making it essential to understand their potential interactive effects on emissions. However, because emissions are species‐specific and highly complex, it can be difficult to generalize how warming and drought may affect VOC production across different environmental and plant‐level contexts (Llusià et al. 2006; Staudt et al. 2016). Furthermore, in situ VOC studies are necessary to aid in our understanding of how climate factors affect emissions from plants in their natural environment (Pierik et al. 2014), as results of greenhouse experiments may differ substantially from field studies (Heinze et al. 2016; Wilschut et al. 2022), but these in situ climate VOC experiments are less common (Midzi et al. 2022).

In this study, our focal study species is tall goldenrod ( *Solidago altissima* ), which is a native forb in old‐field plant communities in the Midwest region of the United States. This species has been previously used in VOC experiments (Morrell and Kessler 2017; Howard et al. 2020; Shiojiri et al. 2021), but to our knowledge, no studies have investigated warming or drought effects on the VOC emissions of this species. *Solidago altissima* and its close relatives also have known ecological importance for plant community succession and plant–insect interactions (Root and Cappuccino 1992; Uriarte 2000; Pisula and Meiners 2010), as they are relatively dominant species in many Midwest plant communities (Eckberg et al. 2023) and interact with numerous generalist to specialist insect species (Maddox and Root 1990; Jobin et al. 1996), making it a good candidate for understanding climate effects on VOC emissions in a natural system. We use an in situ climate change experiment in Michigan, USA to understand how warming, drought, and the combined effects of warming and drought may alter the composition and abundance of VOC emissions for a common species ( *S. altissima* ) in an early successional community.

## 2 Methods

### 2.1 Study Site and Species

The climate treatments in this experiment were applied to plants in situ in the Kellogg Biological Station's Long‐Term Ecological Research site (KBS‐LTER) in Hickory Corners, MI, USA (42.41° N, −85.37° W). This site was under agricultural management until 1989, when management ceased and six replicate fields were re‐established as early successional plant communities. The fields have been maintained in an early successional stage through annual spring burns since 1997, which prevent woody species colonization. Within the KBS‐LTER early successional fields, the most dominant species consist of tall goldenrod ( *Solidago altissima* ), red clover ( *Trifolium pratense* ), timothy grass ( *Phleum pratense* ), and Kentucky bluegrass ( *Poa pratensis* ) (Robertson and Hamilton 2015).

### 2.2 Treatments

Within the KBS‐LTER, a large‐scale climate manipulation experiment known as the Rain‐Exclusion Experiment (REX) began in December 2020. The experiment consists of subplots nested within six separate early successional field replicates, with each subplot receiving a treatment of drought, warming, or their combination, in addition to ambient control subplots. The ambient control subplots used here are open subplots with no treatments applied. Each of the six field replicates contains all four climate treatments (Figure S1A).

Each year since 2021, rainout shelters have been placed above the plant community and their subplots in each of the six early successional fields for 6 weeks to simulate drought during the peak growing season (Figure S1B). The rainout shelters are built with a galvanized steel frame with Lexan clear corrugated roofing, which allows 85%–90% of light in photosynthetically active wavelengths to pass through (Kahmark et al. 2024). Prior to the initiation of drought, all subplots were watered to ensure the plants began the experiment with equal soil moisture. Physiological drought was confirmed with findings of lower soil volumetric water content, lower leaf level gas exchange, lower evapotranspiration rates, and lower GPP in the drought plots (Falvo 2024). Our sampling took place in 2022, and the rainout shelters were placed over the plant communities on 25 June 2022. Temperature is manipulated in this experiment with the use of open‐top chambers (OTCs) built for taller stature plant communities (Welshofer et al. 2018). The OTCs are built with a wooden frame with Lexan polycarbonate sheeting and passively increase air temperatures while allowing for natural levels of light, precipitation, and gas exchange to occur (Marion et al. 1997; Welshofer et al. 2018). The chambers were initially placed on top of the established early successional plant communities in December 2020. OTCs remain on the plots year‐round and are only removed when the annual spring burn occurs; when our sampling took place, the subplots had been continuously warmed for 20 months. Within REX, the open‐top chambers are also nested underneath the rainout shelters to allow for the combination of both warming and drought treatments.

Response variables were measured within 1 m^2^ subplots situated under the footprint of each of the four main treatments used in this study (warmed, drought, warmed + drought, and ambient control; Figure S1). MX2202 HOBO data loggers (Onset Computer Corporation, Bourne, MA) were placed in the subplots to determine the effects of our treatments on 1 m air temperatures. Soil temperature and moisture in the top 15 cm of soil were monitored using Campbell Scientific CS655 probes.

### 2.3 Plant Headspace Collection

From 11 July to 15 July 2022, VOCs were collected from the headspace of *S. altissima* from five of the six fields. We collected VOC samples from one field per day over the course of our consecutive 5‐day sampling period. At the time of sampling, the plants had been in drought for 17–21 days, depending on the field that was sampled (field 1 = 17 days, field 2 = 18 days, field 3 = 19 days, field 4 = 20 days, field 5 = 21 days).

Five plants within each of the four treatments were sampled each day (*n* = 20 plants per day). Plants with any signs of damage, herbivory, or poor health were not selected for VOC collection. However, there were very few plants with obvious signs of herbivory or damage, and *S. altissima* is extremely abundant in our experimental subplots; therefore, our selection of “healthy” plants was not a biased selection toward the healthiest plants in each subplot. The headspace was defined as the area immediately surrounding the top ~30 leaves from each selected plant. This area was contained within a 35 × 32.5 cm nylon oven bag (Jerina turkey bags). On each day, an empty nylon bag was also sampled in situ to serve as a background air control. The top corner of each nylon bag was fitted with an ORBO coconut charcoal filter to allow clean air to enter the bag during sampling (Figure S2). Air was pulled through the bags for 7 h each day (08:30–15:30 h) and onto HayeSep Q VOC traps (volatilecollectiontrap.com) via portable vacuum pumps (IONTIK) at a flow rate of 300 mL/min. Storms on two sampling days cut sampling time to 5 h for Fields 1 and 2 (08:30–13:30 h), but on all sampling days, all treatments in a given field were sampled for the same amount of time. We did not find evidence of the storm strongly affecting VOC composition or abundance (Figure S3).

Once the sampling was complete, the VOC traps were stored in aluminum foil on ice and immediately taken to the laboratory to be processed. Each trap was eluted with 150 μL of dichloromethane, which was pushed through the trap using nitrogen gas. One μL of 500 g/μL tetradecane was then added to each sample as an internal standard. VOCs were analyzed with an Agilent 7890A gas chromatogram (GC) fitted with an Agilent VF‐5 ms column (30 m length, 0.25 mm diameter, 0.25 μm film) with He as the carrier gas and coupled with an Agilent 5975C mass spectrometer (MS). Samples were injected into the GC/MS with an initial temperature of 225°C. The temperature program heated the column from 40°C to 180°C at a rate of 10°C/min, then heated at a rate of 40°C/min until the temperature reached 220°C, which was held for 10 min.

The REX experimental subplots are sampled nondestructively, and therefore no plant material can be taken from within the 1 m^2^ experimental subplots. As such, we did not harvest plant material post‐VOC sampling for a calculation of total biomass sampled. To estimate the biomass sampled per plant, we collected leaves of *S. altissima* from plants immediately outside of each 1m^2^ subplot but still within each treatment. For each treatment, we then fit a linear regression between the collected leaf biomass and leaf length (Table S1). Prior to VOC sampling, we marked the top ~30 leaves of the five *S. altissima* individuals in each subplot and recorded the length of each leaf that was to be sampled during VOC sampling. Using our regression equations, we applied each treatment‐specific regression to the corresponding measured leaf lengths from the sampled VOC plants in order to estimate total leaf biomass per subplot (Table S2). Because the biomass data were estimated on a subplot level and not an individual plant level, we divided the total biomass per subplot by 5 to estimate total plant biomass per individual (Table S2).

### 2.4 Statistical Analysis

The temperature and soil moisture data were analyzed using R (R Core Team 2024). We calculated average air temperature, soil temperature, and soil moisture from 11 July to 15 July to match the VOC sampling time frame. We also calculated monthly averages to determine the beginning of the warming treatment, as well as daily averages from June to July to observe treatment effects for the duration of the drought treatments.

The VOC GC/MS data were first run through the Agilent MassHunter Qualitative Analysis 10.0 program, in which compounds were identified by comparing them to those in the NIST17 (National Institute of Standards and Technology, Gaithersburg, MD) and Adams (Adams 2007) libraries. All further data processing was conducted using R (R Core Team 2024). We normalized the data by dividing the abundance of each compound in each sample by the abundance of the internal standard in each sample. After normalizing the data, the abundance of the internal standard was removed from each sample (as its abundance was now equal to 1 across all samples). To remove any background noise from the sampling procedure, the abundance of each compound found in the nylon bag controls was subtracted from the abundances for each sample. Any abundance that became negative after subtraction, which indicates compounds that were found within the control, was replaced with a zero. Caprolactam, which was present due to the nylon oven bags used for sampling, was also removed from each sample. Samples from field 1 were removed prior to analyses due to a sample processing error (*n* = 24). Samples were also removed prior to analyses if they did not contain the internal standard (*n* = 1) or if the sample had abnormally high abundances, which could indicate plant stress from unseen sources such as herbivory (*n* = 3). After sample removal, each climate treatment had *n* = 18–20 samples. We confirmed that outlier removal did not impact overall results (Table S3). To obtain a final measure of compound abundance/g/h, we divided each individual plant's VOC abundances by its estimated individual biomass, and then by the total number of hours sampled, which was either 7 or 5 h. We also tested for treatment effects on abundance/h, without normalizing by gram of biomass, to look at effects on absolute emissions. We include both biomass per gram and absolute abundance because absolute abundance may be a relevant metric for understanding the cues available to organisms that detect total VOC emissions in the atmosphere, whereas abundance per unit biomass standardizes emissions by plant weight and may be more appropriate for comparing emission rates among plants of different sizes.

To test for differences in air temperature, soil temperature, and soil moisture between treatments, we ran a mixed model for each with climate treatment as a fixed effect and field number as a random effect. These mixed models were conducted using the lmer function from the lmerTest package (Kuznetsova et al. 2017), and we tested all pairwise comparisons using the emmeans package (Lenth 2022).

The compositional differences between climate treatments were investigated using a PERMANOVA (method = Bray‐Curtis, permutations = 999, block = field replicate) via the adonis2 function in the R vegan package (Oskanen et al. 2022). We then ran pairwise comparisons for all climate treatments using the pairwise.adonis2 function. We visualized these compositional differences using a PCoA with Bray‐Curtis distances in the vegan package. We also ran an additional supervised Partial Least Squares Discriminant Analysis (PLS‐DA) using the mixOmics R package (Rohart et al. 2017) to further visualize compositional differences between treatments. To test for an effect of the climate treatments on the abundance of VOCs emitted, we ran a mixed effects model using the lmer function from the lmerTest package in R (Kuznetsova et al. 2017). For the mixed effects model, VOC abundances were transformed via a cubed root transformation to ensure the data fit the assumptions of normality, and field number was included as a random effect.

To test for specific indicator compounds between treatments (i.e., compounds associated with specific climate treatments), we used the multiplatt function (permutations = 999, block = field replicate, max.order = 3) from the indicspecies R package (Cáceres and Legendre 2009). The statistical output from multiplatt provides an indicator value (“stat”) as the test statistic, which measures the association between a compound and a group; this statistic is associated with a *p*‐value. The output also provides the specificity (“A”) and sensitivity (“B”) of that compound to given climate treatments (Table S4). For example, A = 1.0, B = 0.3 would demonstrate that the compound was only found in a single climate treatment, but not all replicates of that treatment. Conversely, A = 0.3, B = 1.0 would demonstrate that the compound was found in all replicates of that treatment but not solely found within that treatment.

To better understand the classification of the 29 compounds identified in the indicator species analyses, we searched known chemical databases for further classification information that was not available from the chemical libraries (Table S5). The databases we included in our search were PubChem (Kim et al. 2023), Pherobase (El‐Sayed 2024), mVOC 4.0 (Lemfack et al. 2018), and the plant‐associated VOC database (PVD; Shao et al. 2024). Compounds were cross‐searched between these databases using their PubChem CID number and the name of the compound. We also performed literature searches on each compound to identify its potential biological function for the plant.

## 3 Results

### 3.1 Abiotic Measurements

**FIGURE 1:**
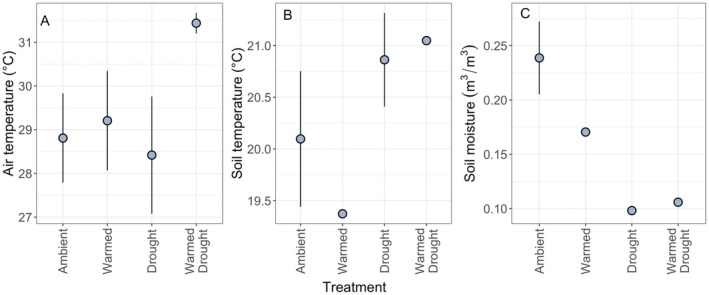
(A) Air temperatures at 1 m above soil level (°C), (B) Soil temperatures integrated across the top 15 cm (°C), and (C) Soil moisture across the top 15 cm (m^3^/m^3^) in all climate treatments from 11 July to 15 July during daytime hours (07:00–19:00). The points and error bars represent mean ± SE (Air temperature: *N* = 4 for all; Soil temperature and moisture: Ambient *n* = 3, drought *n* = 4, warmed *n* = 1, warmed + drought *n* = 1).

**FIGURE 2:**
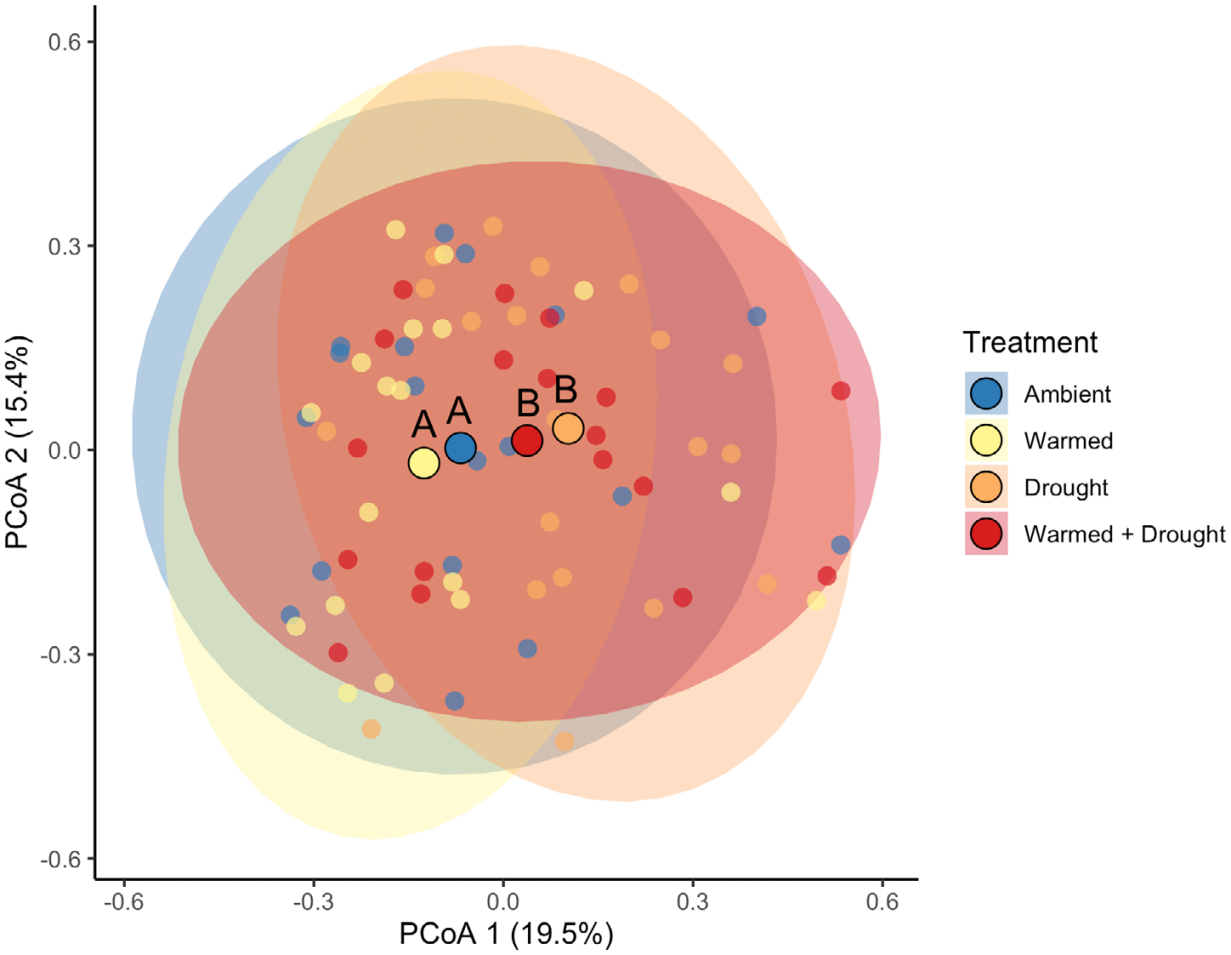
PCoA plot using Bray–Curtis dissimilarity for the VOC composition between climate treatments (ambient, warmed, drought, and warmed + drought). Each point represents the composition of an individual plant, and the ellipses represent the 95% confidence interval. The large, outlined point represents the centroid for each climate treatment, and different letters denote statistical differences in VOC composition from a PERMANOVA.

Warmed treatments (warmed and warmed + drought) began to experience warmer temperatures than nonwarmed treatments (ambient and drought) in June (Figure S4). Air temperatures during the VOC sampling period (11 July–15 July, 07:00–19:00) in the warmed + drought treatment were ~2.5°C warmer than air temperatures in the ambient (*t* = −4.61, *p* < 0.001), warmed (*t* = −3.91, *p* = 0.001), and drought (*t* = −5.29, *p* < 0.001) treatments (Figure 1A). The warmed treatment had lower soil temperatures than drought (*t* = 2.82, *p* = 0.02) and warmed + drought (*t* = 3.13, *p* = 0.01), whereas the ambient treatment had lower soil temperatures than drought (*t* = 2.49, *p* = 0.06) (Figure 2B). All treatments differed from each other in terms of soil moisture, with the ambient and warmed treatments having the highest levels of soil moisture (Figure 1C).

### 3.2 VOC Composition and Abundance

The composition of VOCs in the ambient and warmed treatments differed significantly from the composition of VOCs in the drought (ambient: *F*_1,37_ = 1.67, *p* = 0.04; warmed: *F*_1,37_ = 1.81, *p* = 0.04) and warmed + drought (ambient: *F*_1,35_ = 1.85, *p* = 0.03; warmed: *F*_1,35_ = 1.83, *p* = 0.04) treatments (Figures 2 and S5, Table S3a). The composition did not differ between ambient and warmed (*F*_1,35_ = 0.83, *p* = 0.55) or drought and warmed + drought (*F*_1,37_ = 0.69, *p* = 0.73) treatments (Figures 2 and S5, Table S3a).

**FIGURE 3:**
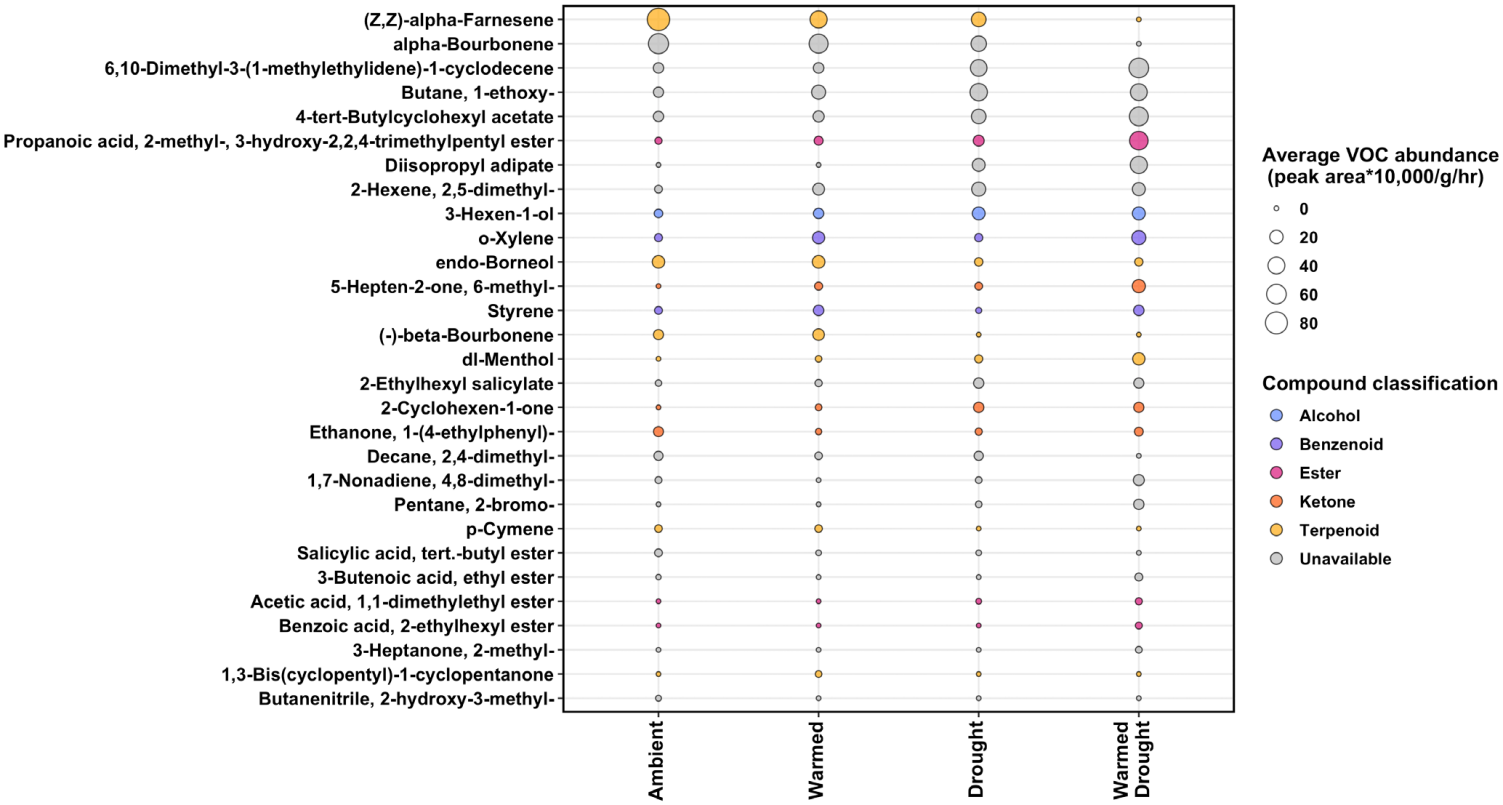
Bubble plot showing the abundance of specific VOCs between each climate treatment. These compounds were selected based on their significant association with at least one climate treatment based on indicator species analysis (Table S4). Compounds are ordered on the y‐axis in terms of decreasing mean abundance. Larger bubbles represent a greater abundance of that compound in that treatment. Compound bubbles are colored by the classification of that compound (Table S5).

With the indicator species analysis, we found 29 compounds to be significantly associated with one or more climate treatments (Figure 3 and Table S4). For example, diisopropyl adipate was significantly associated with the drought and warmed + drought treatment groups and was only found within those treatments (A = 1.00, B = 0.34, stat = 0.59, *p* = 0.002; Table S4). Conversely, p‐cymene was significantly associated with the ambient and warmed treatment groups (A = 1.00, B = 0.19, stat = 0.44, *p* = 0.03; Table S4). However, we did not find differences in the overall abundance of VOCs emitted among treatments, neither for emissions per unit biomass (*F*_3,70_ = 0.34, *p* = 0.80; Figure S6) nor for absolute emissions (*F*_3,70_ = 0.24, *p* = 0.87; Figure S7). Fifteen out of the 29 identified compounds for which we could classify the VOC category included ketones, esters, terpenoids, benzenoids, and alcohols (Figure 3 and Table S5). Six out of the 15 classified compounds were terpenes/terpenoids, whereas the other categories were less common (3 ketones, 3 esters, 2 benzenoids, and 1 alcohol; Table S5). Furthermore, we identified potential functions for 15 of the 29 compounds (Table S6). The majority of these potential functions were related to herbivore defense, whereas the others were related to antifungal, antibacterial, and/or stress responses (Table S6).

## 4 Discussion

We observed differentiation in VOC composition between the nondrought (ambient and warmed) and drought (drought and warmed + drought) treatment groups. Based on this differentiation, the overall composition of VOCs in *S. altissima* appears to be more affected by drought than by warming. We base this conclusion on the fact that there was separation between nondrought and drought treatments, but not between nonwarmed (ambient and drought) and warmed (warmed and warmed + drought) treatments (Figures 2 and S5). There are a few potential explanations for why the drought treatment had a stronger effect on VOC composition than warming.

First, drought may be affecting VOC production more if it is a stronger stressor than the warming treatment; Trowbridge et al. (2019) also found that drought appears to override the effects of warming on *Pinus edulis* emissions. During our sampling period, the drought treatments had lower soil moisture and increased temperatures compared to nondrought treatments (Figure 1). However, only the warmed + drought treatment demonstrated increased air temperatures, whereas our warmed treatment was not different from the ambient or drought treatments in terms of air temperature. The lack of warming in our warmed‐only treatment during our sampling period may have led to a reduced warming effect on overall VOC production. However, we do know that the warmed treatment increased air temperatures prior to our sampling period (Figure S8), and a nearby experiment using the same OTC design demonstrated warming over 7 years (Young et al. 2024), confirming our experimental warming methods as effective. Furthermore, we do not expect to see soil warming with the use of OTCs, as increased biomass due to warming leads to cooling at ground level within the chambers (Young et al. 2024). Therefore, any effects of the warming treatment we found in our indicator species analysis were most likely due to increased air temperatures that occurred prior to our sampling, that is, “pre‐warming.” This effect of prewarming demonstrates that these changes in emissions were likely due to physiological changes in the plant due to prior warming, rather than changes due to concurrent heat affecting the volatilization of compounds. In other words, this prewarming treatment potentially better demonstrates the effects of prior, long‐term warming on plant VOC emissions when compared to plants experiencing current warming, which may show differing effects on emissions due to ongoing changes in plant metabolism and chemical volatilization properties.

Second, we initiated the drought ~3 weeks prior to VOC collection, whereas the plants had been warmed for 20 months leading up to our study. The drought treatment was therefore more recent for the plants and could have led to a strong initial response (Franks et al. 2014). Because the plants had been experiencing warmer temperatures for several months leading up to sampling, the warming may have caused an initial response that we did not capture due to our measurements being collected after several months of warming (Kristensen et al. 2020). As such, our results therefore capture a transient snapshot of plant VOC emissions under experimental climate treatments and do not necessarily represent long‐term responses, especially for the shorter‐term drought treatment. Future studies could conduct longitudinal, multiseason sampling of plant emissions in these climate treatments to better capture variability in emissions and distinguish between immediate and acclimated responses.

Furthermore, although the PERMANOVA demonstrated significant differences between the composition of nondrought and drought treatments, the PCoA shows notable overlap between climate treatments (Figure 2). This demonstrates that the climate treatments may not lead to large, pronounced differentiation in VOC blends; rather, the composition of VOCs may change in more nuanced ways, with a few key compounds being enhanced or suppressed. The indicator species analysis found compounds that were significantly associated with one or more climate treatments, and through literature searches of these compounds, we identified three compounds with potential stress‐related functions: 5‐Hepten‐2‐one, 6‐methyl‐ (warmed + drought treatment); 2‐Ethylhexyl salicylate (drought and warmed treatments); and 3‐Hexen‐1‐ol (drought, warmed, and warmed + drought treatments; Table S6). 5‐Hepten‐2‐one, 6‐methyl‐ has been found to promote programmed cell death (Niu et al. 2024), which may occur due to plant stress. 2‐Ethylhexyl salicylate has been found to protect against UV radiation for desert plants (Matsunaga et al. 2008) and is released under hot temperatures. Finally, 3‐Hexen‐1‐ol can have hyperosmotic stress tolerance functions resulting from decreasing stomatal conductance (Hu et al. 2020) and could aid the plant when experiencing warming and/or drought stress. Additionally, many other compounds were identified as having herbivore defense and antimicrobial properties (Table S6).

Taken together, these results suggest that warming and drought may induce both protective and potentially harmful VOC responses: Some compounds appear to mitigate stress (2‐Ethylhexyl salicylate and 3‐Hexen‐1‐ol), while others may signal stress‐related cell death (5‐Hepten‐2‐one, 6‐methyl‐). We also found declines in compounds associated with defense against pathogens and herbivores, which could further reduce plant fitness under climate stress (Figure 3 and Table S6). Future work could experimentally validate the fitness effects of these compounds, as well as uncover the functions of VOCs that have not yet been described in the literature (i.e., compounds with “NA” in Table S6).

We also found that the overall abundance of VOCs did not differ between treatments, either for abundance per unit biomass or for absolute biomass (Figures S6 and S7). While we hypothesized that the overall abundance of VOCs would increase under warming, we may not be seeing overall differences in abundances due to the nuanced effects of these climate treatments on specific compounds; some compounds or groups of compounds may be enhanced, while others are suppressed, leading to no overall net change in abundances between treatments. However, although not significant, total absolute emissions appear to be higher in the warmed treatment when compared to the other climate treatments (Figure S7), which matches expectations of greater compound abundances under warming (Hartikainen et al. 2009; Peñuelas and Llusià 2003; Yuan et al. 2009). Future studies could further test these ideas by incorporating VOC emissions profiles across multiple species (i.e., community‐level profiles), which may better capture the complexity of natural systems and reveal the effects of climate on emissions at broader ecological scales.

For about half of the 29 compounds (14 out of 29) identified in the indicator species analyses, information on the chemical classification was not available in any database (Table S5). The compounds that did have classification information (15 out of 29) spanned a broad range of VOC categories, which demonstrates that the climate treatments affected multiple VOC types rather than mainly affecting a specific type of VOCs. Based on this information, we found terpenes/terpenoids to be the most common category identified in the indicator species analysis (6 out of 15), showing that this class of compounds may be more sensitive to climate conditions than others. Past studies have also identified terpenes, namely monoterpenes, as having a potential warming stress protective function (Peñuelas and Llusià 2003). However, terpenes/terpenoids are also the largest VOC family (Picazo‐Aragonés et al. 2020), potentially explaining why this classification was the most common. Further classification for the remaining 14 compounds would allow us to more fully determine how climate affects these broad VOC categories. Furthermore, many of the same compounds were missing information across all four databases, highlighting the gap in knowledge for the plant VOCs we identified in this study. Nonetheless, these databases highlight the promise of synthesizing VOC information across resources and studies, and future VOC studies should prioritize their use and contribute to this growing body of knowledge.

Overall, we found effects of both warming and drought on the emission of *S. altissima* VOCs, with specific compounds being more affected than others. Certain compounds, such as 2‐Ethylhexyl salicylate and 3‐Hexen‐1‐ol, have previously been found to have stress‐protectant functions and could similarly be protecting goldenrod here, as they were upregulated under warmed and/or drought treatments. Future studies could further build upon our observational findings to more specifically determine the function of the compounds we identified, which could aid in our understanding of climate effects on important emissions. Furthermore, because it is difficult to generalize climate effects on plant emissions, more coordinated climate change experiments and more fully developed VOC databases are necessary to unravel the mechanisms underlying warming and drought effects on VOC production and emissions.

## Author Contributions

**Kara C. Dobson:** conceptualization (lead), data curation (lead), formal analysis (lead), funding acquisition (equal), investigation (equal), methodology (lead), validation (equal), visualization (lead), writing – original draft (lead), writing – review and editing (equal). **Phoebe L. Zarnetske:** conceptualization (supporting), funding acquisition (equal), investigation (equal), project administration (lead), supervision (lead), validation (equal), writing – review and editing (equal).

## Conflicts of Interest

The authors declare no conflicts of interest.

## Supporting information


**Figure S1:** (A) Experimental design of the climate treatments in the REX at the KBS‐LTER. Each large green square represents a single field replicate (1–6), with the four climate treatment subplots (drought, warmed and drought, ambient, and warmed) present within each field replicate. Five plants were sampled from each treatment subplot. (B) A photograph of a single field replicate, showing a rainout shelter with open‐top chambers nested underneath (left) and an open‐top chamber with no rainout shelter (right) (Photo by Kara Dobson).
**Figure S2:** Diagram of plant headspace VOC collection. Nylon oven bags contained the top 30 leaves of each plant, with an ORBO charcoal filter fitted to the top corner of the bag to pull clean air through. Vacuum pumps pulled air from the plant headspace onto HayeSep Q VOC traps.
**Figure S3:** Compositional (A) and abundance (B) differences between field replicates. Field replicate 2 experienced a storm which cut sampling time short to 5 h, whereas the other field replicates were sampled for 7 h.
**Figure S4:** Average 1 m air temperatures (°C) during daytime hours (07:00–19:00) for each climate treatment (ambient, drought, warmed, and warmed drought) for each month of 2022. Points represent means ± standard error (*n* = 6 ambient and warmed, *n* = 5 drought and warmed drought).
**Figure S5:** Partial least squares discriminant analysis (PLS‐DA) of VOC composition between treatment groups.
**Figure S6:** Average VOC abundance (peak area/g/h) in the ambient, warmed, drought, and warmed + drought treatments. Points represent the mean ± the 95% confidence interval (ambient, warmed, and warmed + drought: *n* = 18, drought: *n* = 20).
**Figure S7:** Average VOC abundance (peak area/h) in the ambient, warmed, drought, and warmed + drought treatments. Points represent the mean ± the 95% confidence interval (ambient, warmed, and warmed + drought: *n* = 18, drought: *n* = 20).
**Figure S8:** Average daily 1 m air temperatures (°C) during daytime hours (07:00–19:00) in the ambient, drought, warmed, and warmed drought treatments from June 1–July 15 2022. Points represent means ± standard error (*n* = 4 for all treatments). Our VOC sampling took place from July 11–15.
**Table S1:** Regression equations between *Solidago altissima* leaf length (cm) and leaf weight (g) for leaves collected from each of the climate treatments (*n* ≈ 60 per treatment). For any given leaf length (*x*), the equation calculates an estimated leaf weight (*y*).
**Table S2:** Average individual plant biomass (g) per field replicate. Averages are based on five plants measured per field replicate.
**Table S3:** Pairwise comparisons of all climate treatments from PERMANOVA model. (a) Model outputs when outliers were removed from the data, and (b) model outputs when using the full dataset.
**Table S4:** Indicator compounds associated with one or more climate treatments. ‘Stat’ represents the indicator value for that compound and group. Value “A” represents the specificity of the compound as an indicator of the group, while value “B” represents the sensitivity of the compound as an indicator. A = 1.0, B = 0.3 would demonstrate that the compound was only found in that specific group, but not all replicates of that group. Conversely, A = 0.3, B = 1.0 would demonstrate that the compound was found in all replicates of that group, but not solely found within that group. Formula: multipatt(ab, voc_transpose$Treatment, max.order = 3, control = how(nperm = 999, blocks = voc_transpose$Rep)).
**Table S5:** Indicator compounds and their associated chemical classification. The databases include PubChem (Kim et al. 2023), Pherobase (El‐Sayed 2024), mVOC 4.0 (Lemfack et al. 2018), and the plant‐associated VOC database (PVD; Shao et al. 2024). The “Final” column combines the classifications from the prior four databases into one final chemical classification determination.
**Table S6:** Indicator compounds and their associated functions identified from literature searches. For compounds with “NA” in the function column, we were not able to find studies in our literature searches that identified the specific functions of that compound.

## Data Availability

Data are available at an Environmental Data Initiative repository: Dobson, K., and P. Zarnetske. (2025). “Data for: Climate Warming and Drought Effects on Volatile Organic Compound Emissions from *Solidago altissima* ver 1.” *Environmental Data Initiative*. https://doi.org/10.6073/pasta/c26998f34bd7fe4fc2e4565f0ce3a184 (Accessed 2025‐04‐07). Code is publicly available via Zenodo: Dobson, K. (2025). dobsonk2/REX_VOCs: Published (published). Zenodo. https://doi.org/10.5281/zenodo.15169944.

## References

[ece372422-bib-0001] Adams, R. P. 2007. Identification of Essential Oil Components by Gas Chromatography/Mass Spectrometry. 4th ed. Allured Publishing Corporation.

[ece372422-bib-0002] Brilli, F. , F. Loreto , and I. Baccelli . 2019. “Exploiting Plant Volatile Organic Compounds (VOCs) in Agriculture to Improve Sustainable Defense Strategies and Productivity of Crops.” Frontiers in Plant Science 10: 264. 10.3389/fpls.2019.00264. 30941152 PMC6434774

[ece372422-bib-0003] Cáceres, M. D. , and P. Legendre . 2009. “Associations Between Species and Groups of Sites: Indices and Statistical Inference.” Ecology 90, no. 12: 3566–3574. 10.1890/08-1823.1. 20120823

[ece372422-bib-0004] Eckberg, J. N. , A. Hubbard , E. T. Schwarz , E. T. Smith , and N. J. Sanders . 2023. “The Dominant Plant Species *Solidago canadensis* Structures Multiple Trophic Levels in an Old‐Field Ecosystem.” Ecosphere 14, no. 1: e4393. 10.1002/ecs2.4393.

[ece372422-bib-0005] El‐Sayed, A. M. 2024. “The Pherobase: Database of Pheromones and Semiochemicals.” [Dataset]. https://www.pherobase.com.

[ece372422-bib-0006] Falvo, G. 2024. “On Radiative Forcing and Land Use Change: Causes, Consequences and Solutions.” Doctoral dissertation, Michigan State University. https://lter.kbs.msu.edu/citations/4158.

[ece372422-bib-0007] Franks, S. J. , J. J. Weber , and S. N. Aitken . 2014. “Evolutionary and Plastic Responses to Climate Change in Terrestrial Plant Populations.” Evolutionary Applications 7, no. 1: 123–139. 10.1111/eva.12112. 24454552 PMC3894902

[ece372422-bib-0008] Hartikainen, K. , A.‐m. Nerg , M. Kivimaenpaa , et al. 2009. “Emissions of Volatile Organic Compounds and Leaf Structural Characteristics of European Aspen (*Populus tremula*) Grown Under Elevated Ozone and Temperature.” Tree Physiology 29, no. 9: 1163–1173. 10.1093/treephys/tpp033. 19448266

[ece372422-bib-0009] Heinze, J. , M. Sitte , A. Schindhelm , J. Wright , and J. Joshi . 2016. “Plant–Soil Feedbacks: A Comparative Study on the Relative Importance of Soil Feedbacks in the Greenhouse Versus the Field.” Oecologia 181, no. 2: 559–569. 10.1007/s00442-016-3591-8. 26922335

[ece372422-bib-0010] Howard, M. M. , J. Kao‐Kniffin , and A. Kessler . 2020. “Shifts in Plant–Microbe Interactions Over Community Succession and Their Effects on Plant Resistance to Herbivores.” New Phytologist 226, no. 4: 1144–1157. 10.1111/nph.16430. 31943213

[ece372422-bib-0011] Hu, S. , Q. Chen , F. Guo , et al. 2020. “(Z)‐3‐Hexen‐1‐ol Accumulation Enhances Hyperosmotic Stress Tolerance in *Camellia sinensis* .” Plant Molecular Biology 103, no. 3: 287–302. 10.1007/s11103-020-00992-2. 32240472

[ece372422-bib-0012] Ibrahim, M. A. , A. Nissinen , N. Prozherina , E. J. Oksanen , and J. K. Holopainen . 2006. “The Influence of Exogenous Monoterpene Treatment and Elevated Temperature on Growth, Physiology, Chemical Content and Headspace Volatiles of Two Carrot Cultivars ( *Daucus carota* L.).” Environmental and Experimental Botany 56, no. 1: 95–107. 10.1016/j.envexpbot.2005.01.006.

[ece372422-bib-0013] IPCC . 2021. Climate Change 2021: The Physical Science Basis. Contribution of Working Group I to the Sixth Assessment Report of the Intergovernmental Panel on Climate Change. Cambridge University Press.

[ece372422-bib-0014] Jobin, A. , U. Schaffner , and W. Nentwig . 1996. “The Structure of the Phytophagous Insect Fauna on the Introduced Weed *Solidago altissima* in Switzerland.” Entomologia Experimentalis et Applicata 79, no. 1: 33–42. 10.1111/j.1570-7458.1996.tb00806.x.

[ece372422-bib-0015] Kahmark, K. , M. Jones , S. Bohm , N. Baker , and G. P. Robertson . 2024. Rainfall Manipulation Shelters for Agricultural Research. Zenodo. 10.5281/ZENODO.10607630.

[ece372422-bib-0016] Kim, S. , J. Chen , T. Cheng , et al. 2023. “PubChem 2023 Update.” Nucleic Acids Research 51, no. D1: D1373–D1380. 10.1093/nar/gkac956. 36305812 PMC9825602

[ece372422-bib-0017] Kramshøj, M. , I. Vedel‐Petersen , M. Schollert , et al. 2016. “Large Increases in Arctic Biogenic Volatile Emissions Are a Direct Effect of Warming.” Nature Geoscience 9, no. 5: 349–352. 10.1038/ngeo2692.

[ece372422-bib-0018] Kreuzwieser, J. , M. Meischner , M. Grün , A. M. Yáñez‐Serrano , L. Fasbender , and C. Werner . 2021. “Drought Affects Carbon Partitioning Into Volatile Organic Compound Biosynthesis in Scots Pine Needles.” New Phytologist 232, no. 5: 1930–1943. 10.1111/nph.17736. 34523149

[ece372422-bib-0019] Kristensen, T. N. , T. Ketola , and I. Kronholm . 2020. “Adaptation to Environmental Stress at Different Timescales.” Annals of the New York Academy of Sciences 1476, no. 1: 5–22. 10.1111/nyas.13974. 30259990

[ece372422-bib-0020] Kuznetsova, A. , P. B. Brockhoff , and R. H. B. Christensen . 2017. “lmerTest Package: Tests in Linear Mixed Effects Models.” Journal of Statistical Software 82, no. 13: 1–26. 10.18637/jss.v082.i13.

[ece372422-bib-0021] Lemfack, M. C. , B.‐O. Gohlke , S. M. T. Toguem , S. Preissner , B. Piechulla , and R. Preissner . 2018. “mVOC 2.0: A Database of Microbial Volatiles.” Nucleic Acids Research 46, no. D1: D1261–D1265. 10.1093/nar/gkx1016. 29106611 PMC5753297

[ece372422-bib-0022] Lenth, R. 2022. *emmeans: Estimated Marginal Means, aka Least‐Squares Means* (Version R Package Version 1.8.3) [Computer Software]. https://CRAN.R‐project.org/package=emmeans.

[ece372422-bib-0023] Llusià, J. , J. Peñuelas , G. A. Alessio , and M. Estiarte . 2006. “Seasonal Contrasting Changes of Foliar Concentrations of Terpenes and Other Volatile Organic Compound in Four Dominant Species of a Mediterranean Shrubland Submitted to a Field Experimental Drought and Warming.” Physiologia Plantarum 127, no. 4: 632–649. 10.1111/j.1399-3054.2006.00693.x.

[ece372422-bib-0024] Loreto, F. , and V. Velikova . 2001. “Isoprene Produced by Leaves Protects the Photosynthetic Apparatus Against Ozone Damage, Quenches Ozone Products, and Reduces Lipid Peroxidation of Cellular Membranes.” Plant Physiology 127, no. 4: 1781–1787. 11743121 PMC133581

[ece372422-bib-0025] Maddox, G. D. , and R. B. Root . 1990. “Structure of the Encounter Between Goldenrod ( *Solidago altissima* ) and Its Diverse Insect Fauna.” Ecology 71, no. 6: 2115–2124. 10.2307/1938625.

[ece372422-bib-0026] Malone, S. C. , A. Simonpietri , W. B. Knighton , and A. M. Trowbridge . 2023. “Drought Impairs Herbivore‐Induced Volatile Terpene Emissions by Ponderosa Pine but Not Through Constraints on Newly Assimilated Carbon.” Tree Physiology 43, no. 6: 938–951. 10.1093/treephys/tpad016. 36762917

[ece372422-bib-0027] Marion, G. M. , G. H. R. Henry , D. W. Freckman , et al. 1997. “Open‐Top Designs for Manipulating Field Temperature in High‐Latitude Ecosystems.” Global Change Biology 3, no. S1: 20–32. 10.1111/j.1365-2486.1997.gcb136.x.

[ece372422-bib-0028] Matsunaga, S. N. , A. B. Guenther , M. J. Potosnak , and E. C. Apel . 2008. “Emission of Sunscreen Salicylic Esters From Desert Vegetation and Their Contribution to Aerosol Formation.” Atmospheric Chemistry and Physics 8, no. 24: 7367–7371. 10.5194/acp-8-7367-2008.

[ece372422-bib-0030] Midzi, J. , D. W. Jeffery , U. Baumann , S. Rogiers , S. D. Tyerman , and V. Pagay . 2022. “Stress‐Induced Volatile Emissions and Signalling in Inter‐Plant Communication.” Plants 11, no. 19: 2566. 10.3390/plants11192566. 36235439 PMC9573647

[ece372422-bib-0031] Morrell, K. , and A. Kessler . 2017. “Plant Communication in a Widespread Goldenrod: Keeping Herbivores on the Move.” Functional Ecology 31, no. 5: 1049–1061. 10.1111/1365-2435.12793.

[ece372422-bib-0032] Müller, C. , and R. R. Junker . 2022. “Chemical Phenotype as Important and Dynamic Niche Dimension of Plants.” New Phytologist 234, no. 4: 1168–1174. 10.1111/nph.18075. 35297052

[ece372422-bib-0033] Niinemets, Ü. 2010. “Mild Versus Severe Stress and BVOCs: Thresholds, Priming and Consequences.” Trends in Plant Science 15, no. 3: 145–153. 10.1016/j.tplants.2009.11.008. 20006534

[ece372422-bib-0035] Niu, J. , M. Xu , X. Zhang , et al. 2024. “6‐Methyl‐5‐Hepten‐2‐One Promotes Programmed Cell Death During Superficial Scald Development in Pear.” Molecular Horticulture 4, no. 1: 32. 10.1186/s43897-024-00107-1. 39187899 PMC11348602

[ece372422-bib-0036] Oskanen, J. , G. Simpson , F. Blanchet , et al. 2022. *vegan: Community Ecology Package* (Version R Package Version 2.6–4) [Computer Software]. https://CRAN.R‐project.org/package=vegan.

[ece372422-bib-0038] Peñuelas, J. , and J. Llusià . 2003. “BVOCs: Plant Defense Against Climate Warming?” Trends in Plant Science 8, no. 3: 105–109. 10.1016/S1360-1385(03)00008-6. 12663219

[ece372422-bib-0039] Perreca, E. , F. Eberl , M. V. Santoro , L. P. Wright , A. Schmidt , and J. Gershenzon . 2022. “Effect of Drought and Methyl Jasmonate Treatment on Primary and Secondary Isoprenoid Metabolites Derived From the MEP Pathway in the White Spruce *Picea glauca* .” International Journal of Molecular Sciences 23, no. 7: 3838. 10.3390/ijms23073838. 35409197 PMC8998179

[ece372422-bib-0040] Picazo‐Aragonés, J. , A. Terrab , and F. Balao . 2020. “Plant Volatile Organic Compounds Evolution: Transcriptional Regulation, Epigenetics and Polyploidy.” International Journal of Molecular Sciences 21, no. 23: 8956. 10.3390/ijms21238956. 33255749 PMC7728353

[ece372422-bib-0041] Pierik, R. , C. L. Ballaré , and M. Dicke . 2014. “Ecology of Plant Volatiles: Taking a Plant Community Perspective.” Plant, Cell & Environment 37, no. 8: 1845–1853. 10.1111/pce.12330. 24689452

[ece372422-bib-0042] Pisula, N. L. , and S. J. Meiners . 2010. “Allelopathic Effects of Goldenrod Species on Turnover in Successional Communities.” American Midland Naturalist 163, no. 1: 161–172. 10.1674/0003-0031-163.1.161.

[ece372422-bib-0043] R Core Team . 2024. *R: A Language and Environment for Statistical Computing* [Computer Software]. R Foundation for Statistical Computing. https://www.R‐project.org/.

[ece372422-bib-0044] Reinecke, A. , I. C. Flaig , Y. M. Lozano , M. C. Rillig , and M. Hilker . 2024. “Drought Induces Moderate, Diverse Changes in the Odour of Grassland Species.” Phytochemistry 221: 114040. 10.1016/j.phytochem.2024.114040. 38428627

[ece372422-bib-0045] Rissanen, K. , J. Aalto , A. Gessler , et al. 2022. “Drought Effects on Volatile Organic Compound Emissions From Scots Pine Stems.” Plant, Cell & Environment 45, no. 1: 23–40. 10.1111/pce.14219. 34723383

[ece372422-bib-0046] Robertson, G. P. , and S. K. Hamilton . 2015. “Long‐Term Ecological Research in Agricultural Landscapes at the Kellogg Biological Station LTER Site: Conceptual and Experimental Framework.” In The Ecology of Agricultural Landscapes: Long‐Term Research on the Path to Sustainability, 1–32. Oxford University Press.

[ece372422-bib-0047] Rohart, F. , B. Gautier , A. Singh , and K.‐A. Lê Cao . 2017. “mixOmics: An R Package for ‘Omics Feature Selection and Multiple Data Integration.” PLoS Computational Biology 13, no. 11: e1005752. 10.1371/journal.pcbi.1005752. 29099853 PMC5687754

[ece372422-bib-0048] Root, R. B. , and N. Cappuccino . 1992. “Patterns in Population Change and the Organization of the Insect Community Associated With Goldenrod.” Ecological Monographs 62, no. 3: 393–420. 10.2307/2937117.

[ece372422-bib-0050] Shao, D. , C. Schlagnhaufer , A. Bandara , et al. 2024. “Plant‐Associated Volatile Organic Compound (VOC) Database (PVD): A Resource Supporting Research on VOCs Produced by Plants and Plant‐Associated Microbes.” PhytoFrontiers™ 4: PHYTOFR‐08‐24‐0088‐A. 10.1094/PHYTOFR-08-24-0088-A.

[ece372422-bib-0051] Sharkey, T. D. , and E. L. Singsaas . 1995. “Why Plants Emit Isoprene.” Nature 374, no. 6525: 769. 10.1038/374769a0.

[ece372422-bib-0052] Sharkey, T. D. , A. E. Wiberley , and A. R. Donohue . 2007. “Isoprene Emission From Plants: Why and How.” Annals of Botany 101, no. 1: 5–18. 10.1093/aob/mcm240. 17921528 PMC2701830

[ece372422-bib-0053] Shiojiri, K. , S. Ishizaki , and Y. Ando . 2021. “Plant–Plant Communication and Community of Herbivores on Tall Goldenrod.” Ecology and Evolution 11, no. 12: 7439–7447. 10.1002/ece3.7575. 34188825 PMC8216902

[ece372422-bib-0054] Staudt, M. , X. Morin , and I. Chuine . 2016. “Contrasting Direct and Indirect Effects of Warming and Drought on Isoprenoid Emissions From Mediterranean Oaks.” Regional Environmental Change 17, no. 7: 2121–2133. 10.1007/s10113-016-1056-6.

[ece372422-bib-0055] Tiiva, P. , J. Tang , A. Michelsen , and R. Rinnan . 2017. “Monoterpene Emissions in Response to Long‐Term Night‐Time Warming, Elevated CO2 and Extended Summer Drought in a Temperate Heath Ecosystem.” Science of the Total Environment 580: 1056–1067. 10.1016/j.scitotenv.2016.12.060. 27989477

[ece372422-bib-0056] Trowbridge, A. M. , P. C. Stoy , H. D. Adams , et al. 2019. “Drought Supersedes Warming in Determining Volatile and Tissue Defenses of Piñon Pine ( *Pinus edulis* ).” Environmental Research Letters 14, no. 6: 065006. 10.1088/1748-9326/ab1493.

[ece372422-bib-0059] Uriarte, M. 2000. “Interactions Between Goldenrod ( *Solidago altissima* L.) and Its Insect Herbivore ( *Trirhabda virgata* ) Over the Course of Succession.” Oecologia 122, no. 4: 521–528. 10.1007/s004420050975. 28308345

[ece372422-bib-0060] Velikova, V. , Z. Várkonyi , M. Szabó , et al. 2011. “Increased Thermostability of Thylakoid Membranes in Isoprene‐Emitting Leaves Probed With Three Biophysical Techniques.” Plant Physiology 157, no. 2: 905–916. 10.1104/pp.111.182519. 21807886 PMC3192565

[ece372422-bib-0061] Welshofer, K. B. , P. L. Zarnetske , N. K. Lany , and L. A. E. Thompson . 2018. “Open‐Top Chambers for Temperature Manipulation in Taller‐Stature Plant Communities.” Methods in Ecology and Evolution 9, no. 2: 254–259. 10.1111/2041-210X.12863.

[ece372422-bib-0062] Wilschut, R. A. , J. R. De Long , S. Geisen , et al. 2022. “Combined Effects of Warming and Drought on Plant Biomass Depend on Plant Woodiness and Community Type: A Meta‐Analysis.” Proceedings of the Royal Society B: Biological Sciences 289, no. 1984: 20221178. 10.1098/rspb.2022.1178. PMC9533002 36196543

[ece372422-bib-0063] Young, M. L. , K. C. Dobson , M. D. Hammond , and P. L. Zarnetske . 2024. “Plant Community Responses to the Individual and Interactive Effects of Warming and Herbivory Across Multiple Years.” Ecology 105, no. 11: e4441. 10.1002/ecy.4441. 39363508

[ece372422-bib-0064] Yuan, J. S. , S. J. Himanen , J. K. Holopainen , F. Chen , and C. N. Stewart . 2009. “Smelling Global Climate Change: Mitigation of Function for Plant Volatile Organic Compounds.” Trends in Ecology & Evolution 24, no. 6: 323–331. 10.1016/j.tree.2009.01.012. 19324451

