# Supplementary material for: Volatile Organic Compound Emissions From Solidago altissima Under Experimental Warming and Drought
Source: Ecol Evol. 2025 Nov 11;15(11):e72422. doi: 10.1002/ece3.72422 (PMC12603385; doi:10.1002/ece3.72422)
Supplement: Supplementary file 1 — Figure S1: (A) Experimental design of the climate treatments in the REX at the KBS‐LTER. Each large green square represents a single field replicate (1–6), with the four climate treatment subplots (drought, warmed and drought, ambient, and warmed) present within each field replicate. Five plants were sampled from each treatment subplot. (B) A photograph of a single field replicate, showing a rainout shelter with open‐top chambers nested underneath (left) and an open‐top chamber with no rainout shelter (right) (Photo by Kara Dobson). Figure S2: Diagram of plant headspace VOC collection. Nylon oven bags contained the top 30 leaves of each plant, with an ORBO charcoal filter fitted to the top corner of the bag to pull clean air through. Vacuum pumps pulled air from the plant headspace onto HayeSep Q VOC traps. Figure S3: Compositional (A) and abundance (B) differences between field replicates. Field replicate 2 experienced a storm which cut sampling time short to 5 h, whereas the other field replicates were sampled for 7 h. Figure S4: Average 1 m air temperatures (°C) during daytime hours (07:00–19:00) for each climate treatment (ambient, drought, warmed, and warmed drought) for each month of 2022. Points represent means ± standard error (n = 6 ambient and warmed, n = 5 drought and warmed drought). Figure S5: Partial least squares discriminant analysis (PLS‐DA) of VOC composition between treatment groups. Figure S6: Average VOC abundance (peak area/g/h) in the ambient, warmed, drought, and warmed + drought treatments. Points represent the mean ± the 95% confidence interval (ambient, warmed, and warmed + drought: n = 18, drought: n = 20). Figure S7: Average VOC abundance (peak area/h) in the ambient, warmed, drought, and warmed + drought treatments. Points represent the mean ± the 95% confidence interval (ambient, warmed, and warmed + drought: n = 18, drought: n = 20). Figure S8: Average daily 1 m air temperatures (°C) during daytime hours (07:00–19:00) in the ambient, [file ECE3-15-e72422-s001.docx]

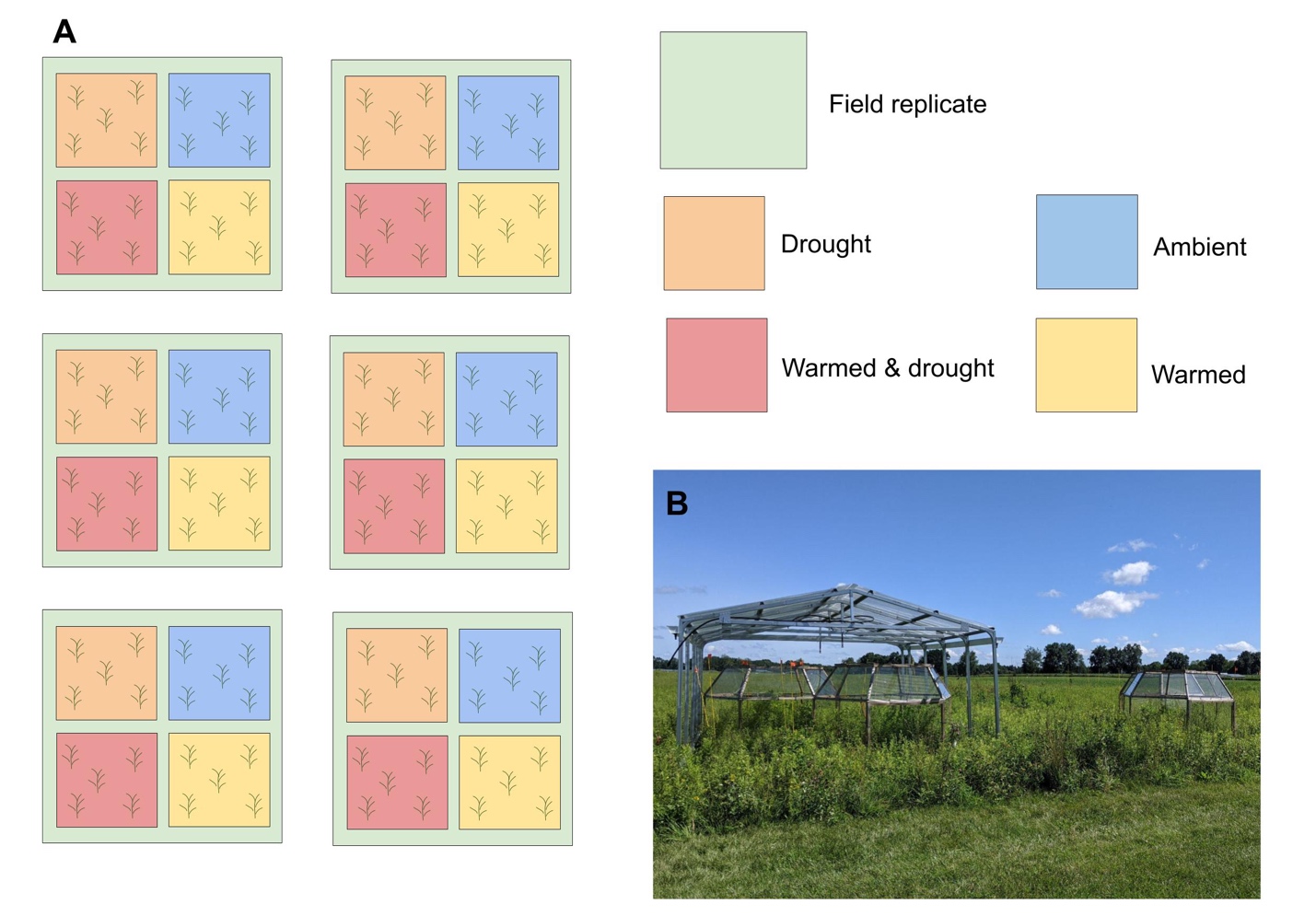


**Figure S1.** (A) Experimental design of the climate treatments in the REX at the KBS-LTER. Each large green square represents a single field replicate (1-6), with the four climate treatment subplots (drought, warmed & drought, ambient, and warmed) present within each field replicate. Five plants were sampled from each treatment subplot. (B) A photo of a single field replicate, showing a rainout shelter with open-top chambers nested underneath (left) and an open-top chamber with no rainout shelter (right) (Photo by Kara Dobson).


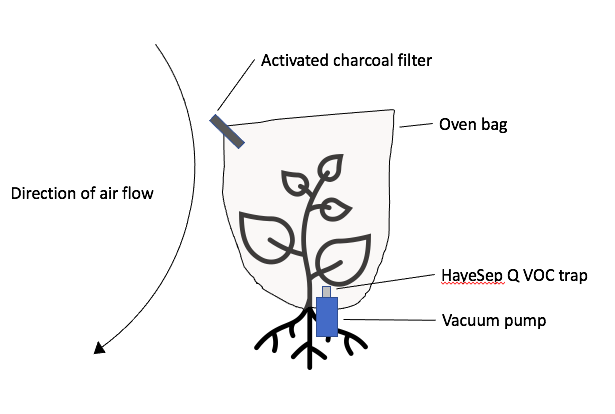


**Figure S2.** Diagram of plant headspace VOC collection. Nylon oven bags contained the top 30 leaves of each plant, with an ORBO charcoal filter fitted to the top corner of the bag to pull clean air through. Vacuum pumps pulled air from the plant headspace onto HayeSep Q VOC traps.


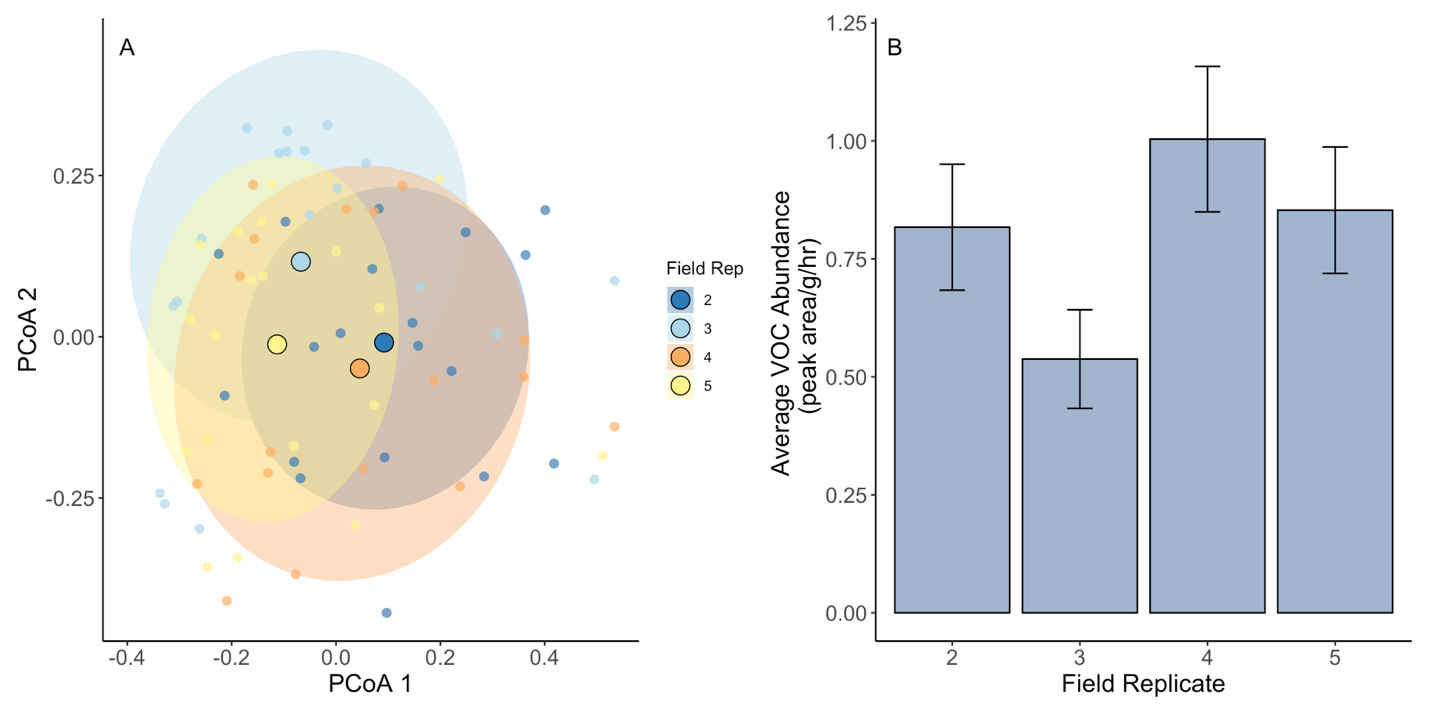


**Figure S3.** Compositional (A) and abundance (B) differences between field replicates. Field replicate 2 experienced a storm which cut sampling time short to 5 hours, whereas the other field replicates were sampled for 7 hours.


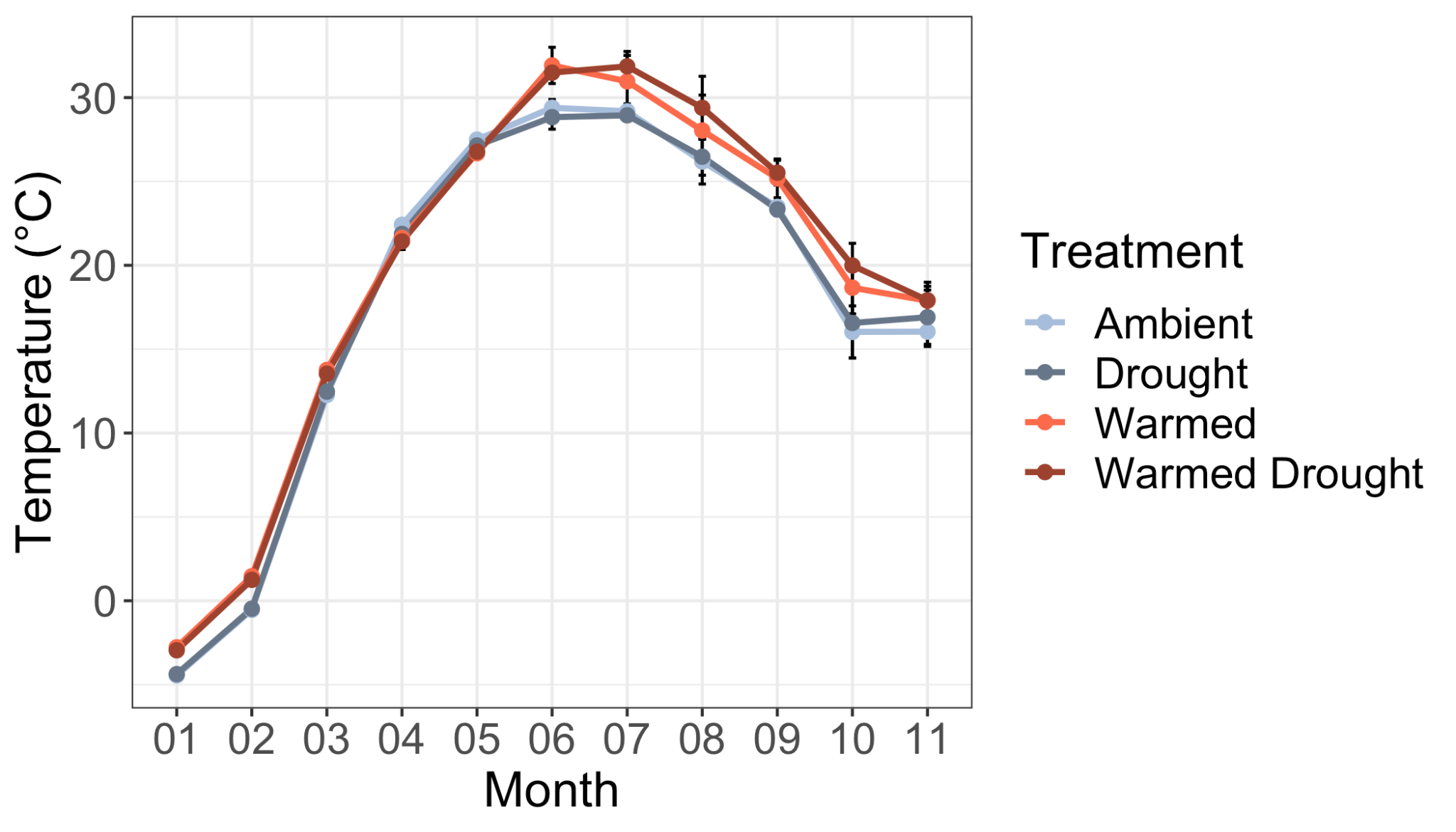


**Figure S4.** Average 1m air temperatures (°C) during daytime hours (07:00-19:00) for each climate treatment (ambient, drought, warmed, and warmed drought) for each month of 2022. Points represent means ± standard error (n = 6 ambient and warmed, n = 5 drought and warmed drought).


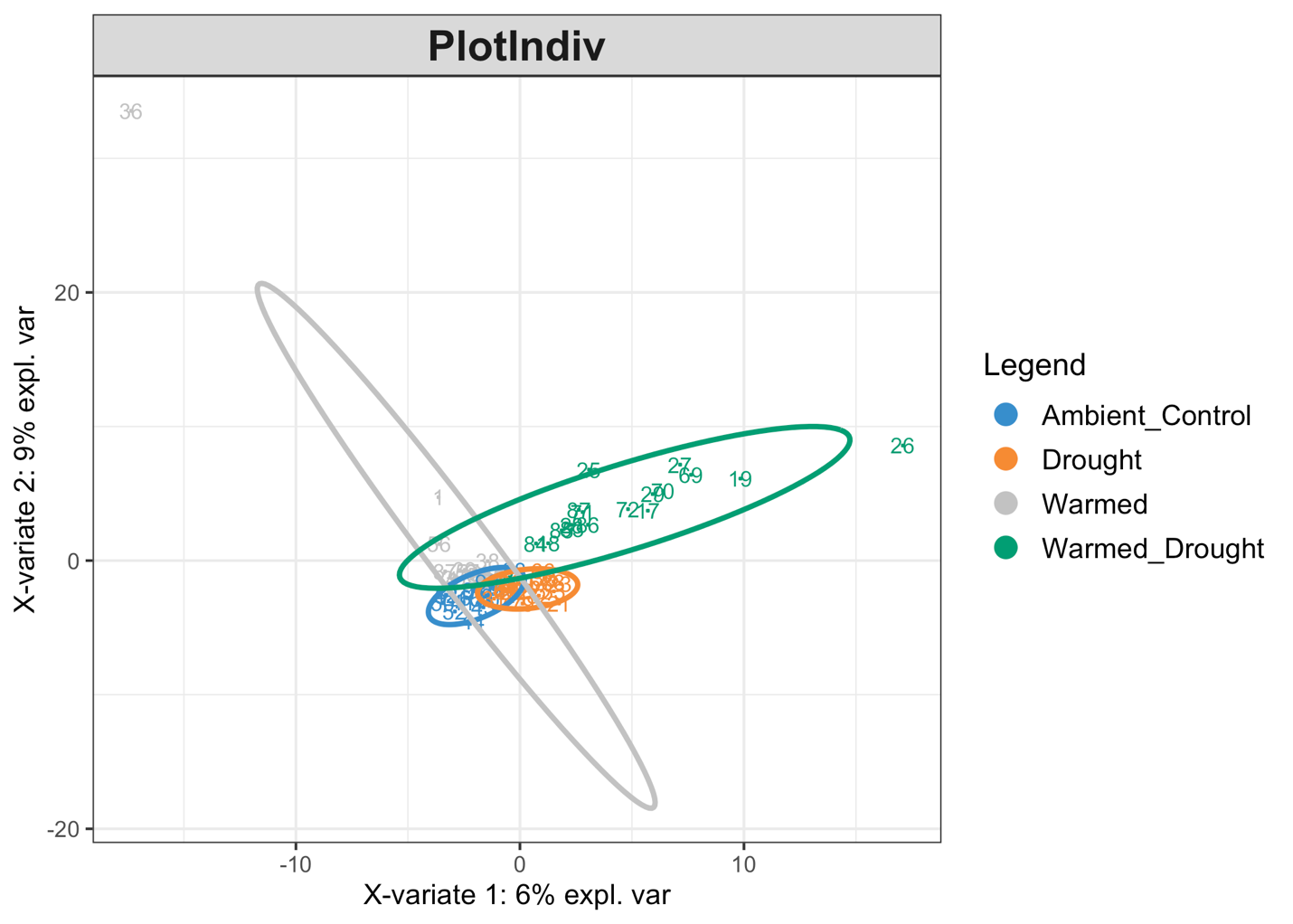


**Figure S5.** Partial least squares discriminant analysis (PLS-DA) of VOC composition between treatment groups.


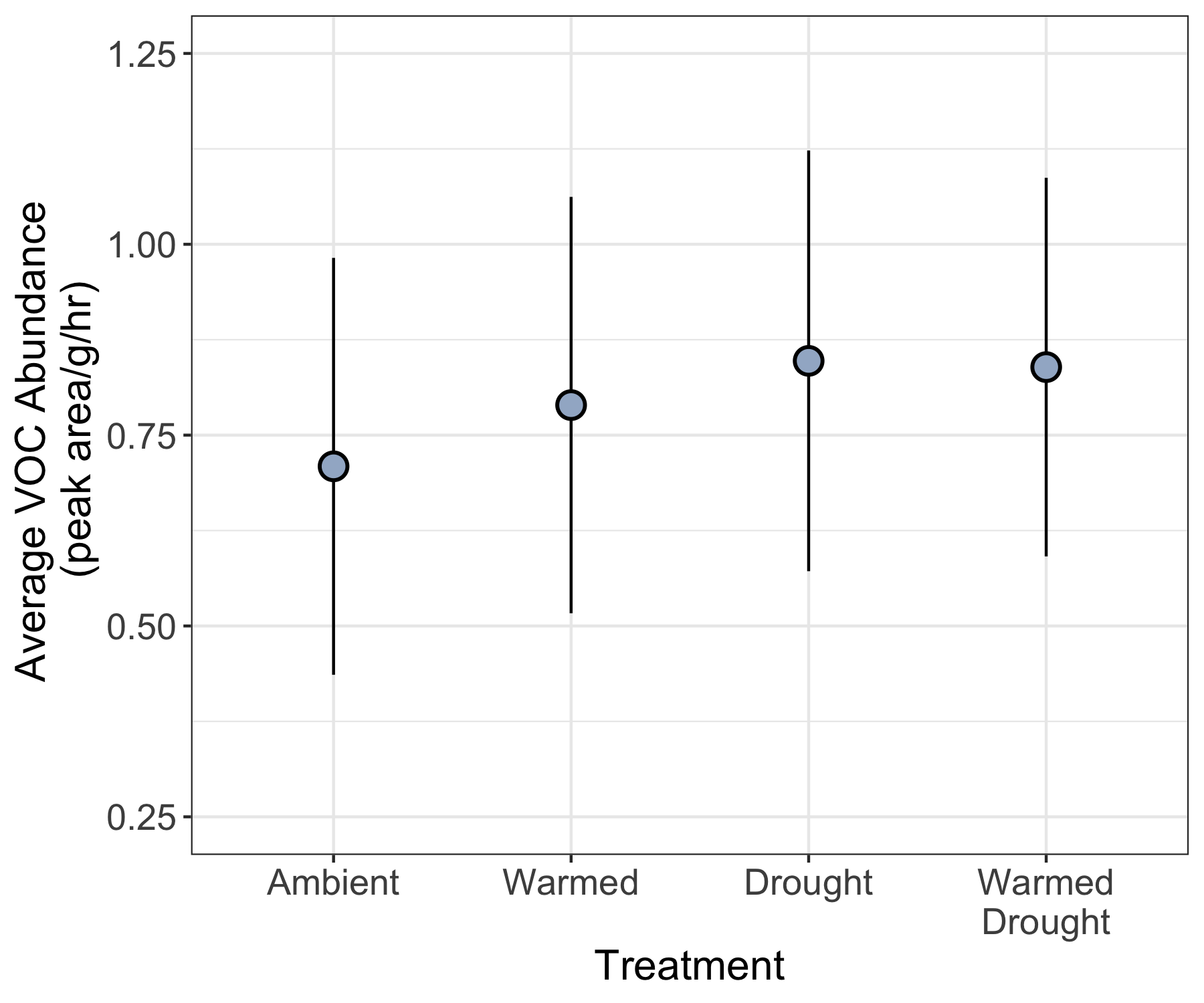


**Figure S6.** Average VOC abundance (peak area/g/hour) in the ambient, warmed, drought, and warmed + drought treatments. Points represent the mean ± the 95% confidence interval (ambient, warmed, and warmed + drought: n = 18, drought: n = 20).


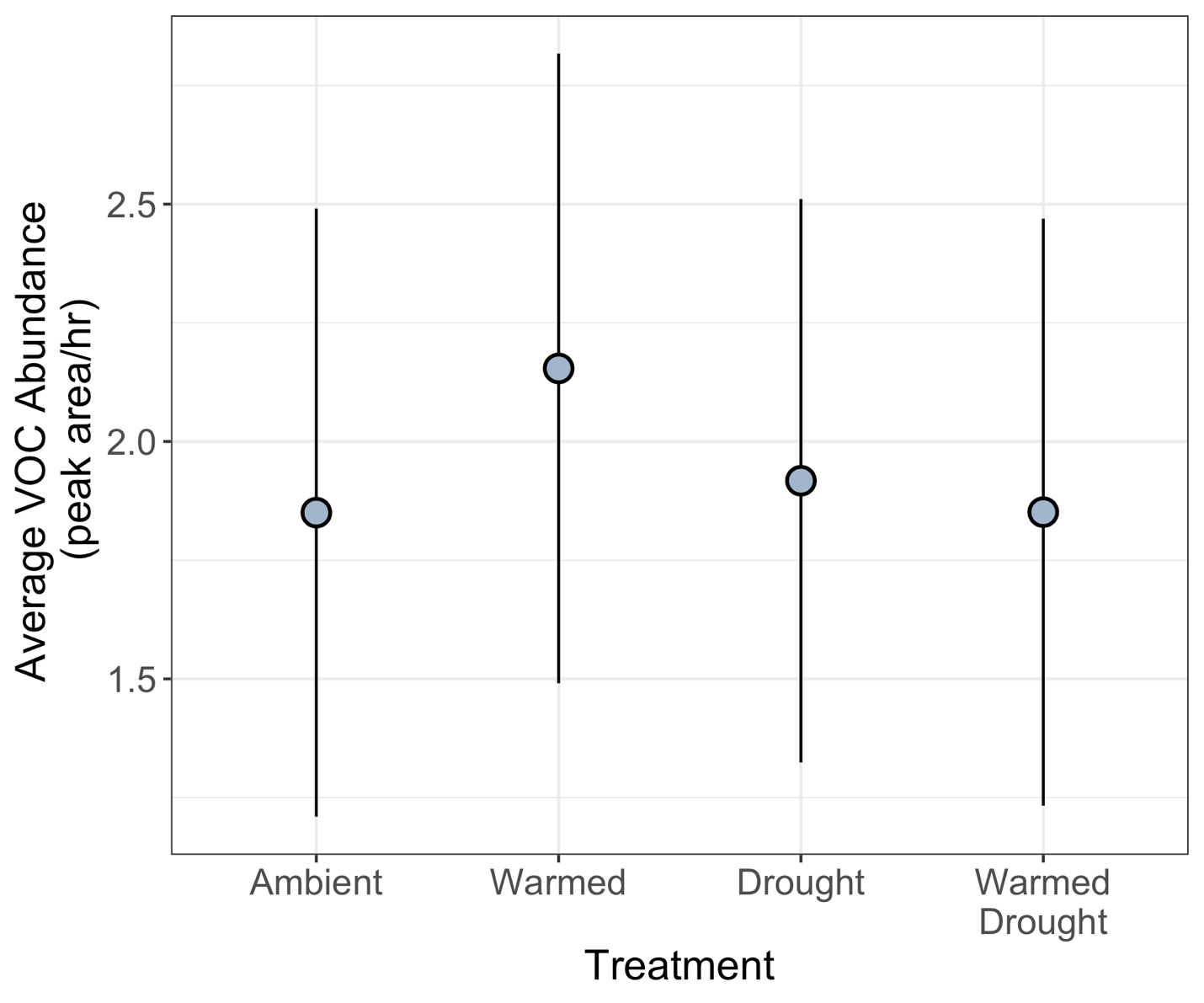


**Figure S7.** Average VOC abundance (peak area/hour) in the ambient, warmed, drought, and warmed + drought treatments. Points represent the mean ± the 95% confidence interval (ambient, warmed, and warmed + drought: n = 18, drought: n = 20).


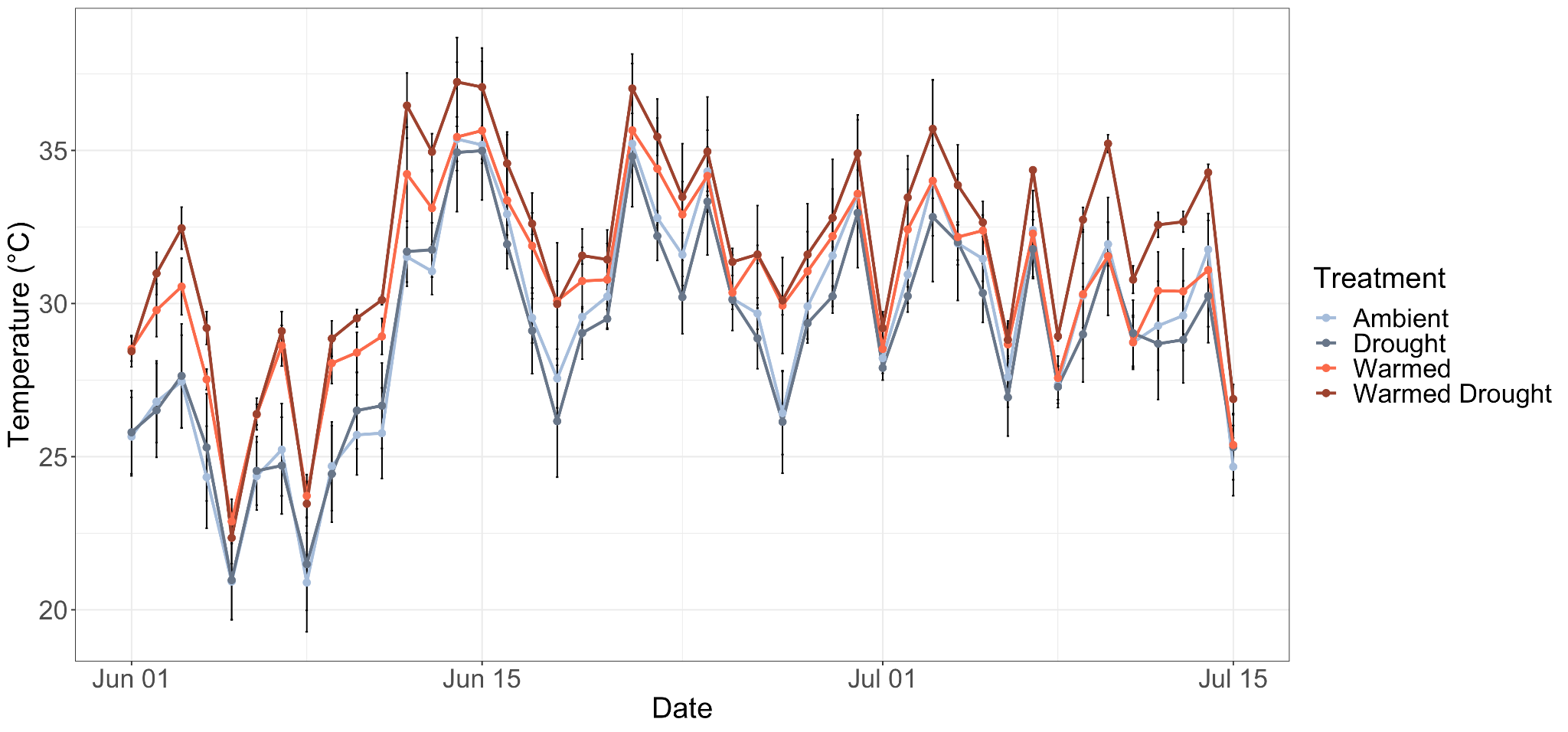


**Figure S8.** Average daily 1m air temperatures (°C) during daytime hours (07:00-19:00) in the ambient, drought, warmed, and warmed drought treatments from June 1- July 15 2022. Points represent means ± standard error (n = 4 for all treatments). Our VOC sampling took place from July 11-15.

**Table S1.** Regression equations between *Solidago altissima* leaf length (cm) and leaf weight (g) for leaves collected from each of the climate treatments (n ≈ 60 per treatment). For any given leaf length (x), the equation calculates an estimated leaf weight (y).

| **Treatment** | **Equation** |
| --- | --- |
| Ambient | y = 0.0217x - 0.0589 |
| Warmed | y = 0.0235x - 0.0654 |
| Drought | y = 0.0192x - 0.0458 |
| Warmed & Drought | y = 0.0221x - 0.0653 |

**Table S2.** Average individual plant biomass (g) per field replicate. Averages are based on five plants measured per field replicate.

| **Treatment** | **Field replicate** | **Average biomass per replicate (g)** | **Standard deviation (g)** |
| --- | --- | --- | --- |
| Ambient | 2 | 3.52 | 0.97 |
|  | 3 | 2.52 | 0.36 |
|  | 4 | 2.31 | 0.48 |
|  | 5 | 2.61 | 0.44 |
| Drought | 2 | 2.30 | 0.42 |
|  | 3 | 2.59 | 0.60 |
|  | 4 | 2.09 | 0.25 |
|  | 5 | 2.34 | 0.33 |
| Warmed | 2 | 3.11 | 0.41 |
|  | 3 | 2.57 | 0.40 |
|  | 4 | 3.15 | 0.22 |
|  | 5 | 2.36 | 0.22 |
| Warmed Drought | 2 | 2.55 | 0.50 |
|  | 3 | 2.78 | 0.79 |
|  | 4 | 1.55 | 0.12 |
|  | 5 | 1.94 | 0.38 |

**Table S3.** Pairwise comparisons of all climate treatments from PERMANOVA model. (a) Model outputs when outliers were removed from the data, and (b) model outputs when using the full dataset.

| **(a) Outliers removed** | | | | | | | | | |
| --- | --- | --- | --- | --- | --- | --- | --- | --- | --- |
| **Treatment comparison** | **DF** | **Sum of Sqs** | | | **R^2^** | **F-Value** | | | **P-Value** |
| Warmed vs Ambient  Treatment  Residual  Total | 1  34  35 | 0.22  9.07  9.29 | | | 0.02  0.98  1.00 | 0.83 | | | 0.55 |
| Warmed vs Warmed Drought  Treatment  Residual  Total | 1  34  35 | 0.49  9.08  9.56 | | | 0.05  0.95  1.00 | 1.83 | | | **0.04** |
| Warmed vs Drought  Treatment  Residual  Total | 1  36  37 | 0.49  9.79  10.28 | | | 0.05  0.95  1.00 | 1.91 | | | **0.04** |
| Ambient vs Warmed Drought  Treatment  Residual  Total | 1  34  35 | 0.49  9.06  9.55 | | | 0.05  0.95  1.00 | 1.85 | | | **0.03** |
| Ambient vs Drought  Treatment  Residual  Total | 1  36  37 | 0.45  9.77  10.22 | | | 0.04  9.56  1.00 | 1.67 | | | **0.04** |
| Warmed Drought vs Drought  Treatment  Residual  Total | 1  36  37 | 0.19  9.77  9.96 | | | 0.02  0.98  1.00 | 0.69 | | | 0.73 |
| **(b) All data** | | | | | | | | | |
| **Treatment comparison** | **DF** | | **Sum of Sqs** | **R^2^** | | | **F-Value** | **P-Value** | |
| Warmed vs Ambient  Treatment  Residual  Total | 1  36  37 | | 0.22  10.28  10.49 | 0.02  0.98  1.00 | | | 0.77 | 0.62 | |
| Warmed vs Warmed Drought  Treatment  Residual  Total | 1  35  36 | | 0.50  9.66  10.16 | 0.05  0.95  1.00 | | | 1.82 | **0.05** | |
| Warmed vs Drought  Treatment  Residual  Total | 1  37  38 | | 0.52  10.37  10.89 | 0.05  0.95  1.00 | | | 1.85 | **0.03** | |
| Ambient vs Warmed Drought  Treatment  Residual  Total | 1  35  36 | | 0.50  9.68  10.17 | 0.05  0.95  1.00 | | | 1.80 | **0.02** | |
| Ambient vs Drought  Treatment  Residual  Total | 1  37  38 | | 0.46  10.39  10.85 | 0.04  0.96  1.00 | | | 1.65 | **0.05** | |
| Warmed Drought vs Drought  Treatment  Residual  Total | 1  36  37 | | 0.19  9.77  9.96 | 0.02  0.98  1.00 | | | 0.69 | 0.70 | |

**Table S4.** Indicator compounds associated with one or more climate treatments. ‘Stat’ represents the indicator value for that compound and group. Value ’A’ represents the specificity of the compound as an indicator of the group, while value ‘B’ represents the sensitivity of the compound as an indicator. A=1.0, B=0.3 would demonstrate that that compound was only found in that specific group, but not all replicates of that group. Conversely, A=0.3, B=1.0 would demonstrate that that compound was found in all replicates of that group, but not solely found within that group. Formula: multipatt(ab, voc_transpose$Treatment, max.order=3, control = how(nperm=999, blocks=voc_transpose$Rep)).

| **Compound** | **Group** | **A** | **B** | **Stat** | **P-value** |
| --- | --- | --- | --- | --- | --- |
| Ethanone, 1-(4-ethylphenyl)- | Ambient | 0.52 | 0.50 | 0.51 | 0.028 |
| Salicylic acid, tert.-butyl ester | Ambient | 0.84 | 0.28 | 0.48 | 0.003 |
| Butanenitrile, 2-hydroxy-3-methyl- | Ambient | 1.00 | 0.17 | 0.41 | 0.014 |
| 1,3-Bis(cyclopentyl)-1-cyclopentanone | Warmed | 1.00 | 0.22 | 0.47 | 0.005 |
| Propanoic acid, 2-methyl-, 3-hydroxy-2,2,4-trimethylpentyl ester | Warmed Drought | 0.75 | 0.50 | 0.61 | 0.001 |
| 1,7-Nonadiene, 4,8-dimethyl- | Warmed Drought | 0.82 | 0.39 | 0.57 | 0.001 |
| 5-Hepten-2-one, 6-methyl- | Warmed Drought | 0.78 | 0.33 | 0.51 | 0.004 |
| dl-Menthol | Warmed Drought | 0.80 | 0.28 | 0.47 | 0.007 |
| Pentane, 2-bromo- | Warmed Drought | 0.90 | 0.22 | 0.45 | 0.004 |
| 3-Heptanone, 2-methyl- | Warmed Drought | 1.00 | 0.17 | 0.41 | 0.045 |
| Benzoic acid, 2-ethylhexyl ester | Warmed Drought | 1.00 | 0.17 | 0.41 | 0.044 |
| 3-Butenoic acid, ethyl ester | Warmed Drought | 0.97 | 0.17 | 0.40 | 0.049 |
| Acetic acid, 1,1-dimethylethyl ester | Warmed Drought | 0.83 | 0.17 | 0.37 | 0.028 |
| Decane, 2,4-dimethyl- | Ambient & Drought | 0.83 | 0.37 | 0.55 | 0.015 |
| endo-Borneol | Ambient & Warmed | 0.83 | 0.33 | 0.53 | 0.015 |
| (Z,Z)-alpha-Farnesene | Ambient & Warmed | 0.82 | 0.33 | 0.52 | 0.029 |
| p-Cymene | Ambient & Warmed | 1.00 | 0.19 | 0.44 | 0.027 |
| (-)-beta-Bourbonene | Ambient & Warmed | 1.00 | 0.19 | 0.44 | 0.050 |
| 4-tert-Butylcyclohexyl acetate | Drought & Warmed Drought | 0.80 | 0.61 | 0.70 | 0.006 |
| 6,10-Dimethyl-3-(1-methylethylidene)-1-cyclodecene | Drought & Warmed Drought | 0.85 | 0.47 | 0.63 | 0.024 |
| 2-Ethylhexyl salicylate | Drought & Warmed Drought | 0.87 | 0.42 | 0.61 | 0.006 |
| Diisopropyl adipate | Drought & Warmed Drought | 1.00 | 0.34 | 0.59 | 0.002 |
| 2-Cyclohexen-1-one | Drought & Warmed Drought | 0.94 | 0.26 | 0.50 | 0.007 |
| o-Xylene | Warmed & Warmed Drought | 0.87 | 0.42 | 0.60 | 0.001 |
| Styrene | Warmed & Warmed Drought | 0.85 | 0.42 | 0.60 | 0.011 |
| alpha-Bourbonene | Ambient & Drought & Warmed | 1.00 | 0.43 | 0.66 | 0.005 |
| 2-Hexene, 2,5-dimethyl- | Drought & Warmed & Warmed Drought | 0.96 | 0.52 | 0.70 | 0.002 |
| 3-Hexen-1-ol | Drought & Warmed & Warmed Drought | 0.92 | 0.50 | 0.68 | 0.048 |
| Butane, 1-ethoxy- | Drought & Warmed & Warmed Drought | 0.93 | 0.48 | 0.67 | 0.026 |

**Table S5.** Indicator compounds and their associated chemical classification. The databases include PubChem (Kim et al. 2023), Pherobase (El-Sayed, 2024), mVOC 4.0 (Lemfack et al., 2018), and the plant-associated VOC database (PVD; Shao et al., 2024). The “Final” column combines the classifications from the prior four databases into one final chemical classification determination.

| **Compound** | **PubChem CID** | **PubChem** | **Pherobase** | | **mVOC 4.0** | **PVD** | **Final** |
| --- | --- | --- | --- | --- | --- | --- | --- |
| Ethanone, 1-(4-ethylphenyl)- | 13642 |  |  | Ketone | | Ketone | Ketone |
| Salicylic acid, tert.-butyl ester | 11424104 |  |  |  | |  |  |
| Butanenitrile, 2-hydroxy-3-methyl- | 11126188 |  |  |  | |  |  |
| 1,3-Bis(cyclopentyl)-1-cyclopentanone | 558566 |  |  |  | |  | *Terpene |
| Propanoic acid, 2-methyl-, 3-hydroxy-2,2,4-trimethylpentyl ester | 6490 |  |  | Ester | |  | Ester |
| 1,7-Nonadiene, 4,8-dimethyl- | 534956 |  |  |  | |  |  |
| 5-Hepten-2-one, 6-methyl- | 9862 | Ketone | Ketone | Ketone | | Ketone | Ketone |
| dl-Menthol | 1254 |  |  | Terpene | | Terpenoid | Terpenoid |
| Pentane, 2-bromo- | 7890 |  |  |  | |  |  |
| 3-Heptanone, 2-methyl- | 25611 |  |  |  | |  |  |
| Benzoic acid, 2-ethylhexyl ester | 94310 |  |  |  | | Ester | Ester |
| 3-Butenoic acid, ethyl ester | 74172 |  |  |  | |  |  |
| Acetic acid, 1,1-dimethylethyl ester | 10908 | Esters |  |  | |  | Ester |
| Decane, 2,4-dimethyl- | 520357 | Aliphatic |  |  | |  |  |
| endo-Borneol | 64685 | Alcohols and Polyols |  |  | | Terpenoid | Terpenoid |
| (Z,Z)-alpha-Farnesene | 5317320 |  |  |  | |  | *Terpene |
| p-Cymene | 7463 | Isoprenoid, monoterpene |  | Terpene | | Terpenoid | Terpene |
| (-)-beta-Bourbonene | 62566 | Isoprenoid, sesquiterpene |  | Terpene | | Sesqui-  terpenoid | Terpene |
| 4-tert-Butylcyclohexyl acetate | 36081 |  |  |  | |  |  |
| 6,10-Dimethyl-3-(1-methylethylidene)-1-cyclodecene | 5367423 |  |  |  | |  |  |
| 2-Ethylhexyl salicylate | 8364 |  |  |  | |  |  |
| Diisopropyl adipate | 23368 |  |  |  | |  |  |
| 2-Cyclohexen-1-one | 13594 | Ketone | Ketone | Ketone | |  | Ketone |
| o-Xylene | 7237 |  |  | Benzenoid | | Benzenoid | Benzenoid |
| Styrene | 7501 |  |  | Benzenoid | | Benzenoid | Benzenoid |
| alpha-Bourbonene | 530816 |  |  |  | |  |  |
| 2-Hexene, 2,5-dimethyl- | 18853 |  |  |  | |  |  |
| 3-Hexen-1-ol | 5284503 | Fatty alcohol |  | Alcohol | | Alcohol | Alcohol |
| Butane, 1-ethoxy- | 12355 |  |  |  | |  |  |

* Identified through literature; classification information for this compound was not present in any database

**Table S6.** Indicator compounds and their associated functions identified from literature searches. For compounds with “NA” in the function column, we were not able to find studies in our literature searches that identified the specific functions of that compound.

| **Compound** | **Potential function(s)** | **Studies** |
| --- | --- | --- |
| Ethanone, 1-(4-ethylphenyl)- | Parasitoid attraction  Herbivore repellent  Antifungal | Huang et al. 2022 *Pest Manag Sci*  Acheampong et al. 2024 *Sci Afr*  Nagrale et al. 2022 *Eur J Plant Pathol* |
| Salicylic acid, tert.-butyl ester | NA |  |
| Butanenitrile, 2-hydroxy-3-methyl- | NA |  |
| 1,3-Bis(cyclopentyl)-1-cyclopentanone | NA |  |
| Propanoic acid, 2-methyl-, 3-hydroxy-2,2,4-trimethylpentyl ester | NA |  |
| 1,7-Nonadiene, 4,8-dimethyl- | Herbivore repellent | Liu et al. 2023 *Chin J Appl Entomol* |
| 5-Hepten-2-one, 6-methyl- | Programmed cell death (fruit scald) | Niu et al. 2024 *Mol Hortic*  Hui et al. 2016 *Postharvest Biol Technol*  Whitaker & Saftner 2000 *J Agric Food Chem* |
| dl-Menthol | Antifungal, antibacterial, insect repellent | Kamatou et al. 2013 *Phytochemistry* |
| Pentane, 2-bromo- | NA |  |
| 3-Heptanone, 2-methyl- | NA |  |
| Benzoic acid, 2-ethylhexyl ester | NA |  |
| 3-Butenoic acid, ethyl ester | NA |  |
| Acetic acid, 1,1-dimethylethyl ester | Antifungal  Biofilm production | Sholberg 2009 *Fresh Produce*  Chen et al. 2015 *MBio* |
| Decane, 2,4-dimethyl- | NA |  |
| endo-Borneol | Antifungal  Antibacterial | Li et al. 2023 *Arab J Chem*  Ma et al. 2023 *Biomed Pharmacother* |
| (Z,Z)-alpha-Farnesene | Antifungal | Shahiri Tabarestani et al. 2016 *Mycol Iran* |
| p-Cymene | Antimicrobial | Marchese et al. 2017 *Materials* |
| (-)-beta-Bourbonene | Larvicide | Kosgei et al. 2014 *Int J Biol Chem Sci* |
| 4-tert-Butylcyclohexyl acetate | Herbivore repellent | Sánchez et al. 2023 (preprint) *Research Square* |
| 6,10-Dimethyl-3-(1-methylethylidene)-1-cyclodecene | NA |  |
| 2-Ethylhexyl salicylate | Pesticide  UV absorption, secondary organic aerosol formation | Yang et al. 2009 *HortScience*  Matsunaga et al. 2008 *Atmos Chem Phys* |
| Diisopropyl adipate | NA |  |
| 2-Cyclohexen-1-one | Antibacterial | Hamada and Gomi 2024 *J Microorg Control* |
| o-Xylene | Herbivore repellent | Chen et al. 2017 *F Physiol* |
| Styrene | Herbivore repellent | Azeem et al. 2013 *J Chem Ecol* |
| alpha-Bourbonene | NA |  |
| 2-Hexene, 2,5-dimethyl- | NA |  |
| 3-Hexen-1-ol | Hyperosmotic stress tolerance | Hu et al. 2020 *Plant Mol Biol* |
| Butane, 1-ethoxy- | NA |  |
